# Simultaneous mating disruption of two moth pests of the vineyard (*Lobesia botrana* and *Cryptoblabes gnidiella*) through a biodegradable sex pheromone dispenser

**DOI:** 10.1007/s11356-024-33980-w

**Published:** 2024-06-24

**Authors:** Renato Ricciardi, Livia De Fazi, Giordana D’Anna, Francesco Savino, Edith Ladurner, Andrea Iodice, Giovanni Benelli, Andrea Lucchi

**Affiliations:** 1 Department of Agriculture, Food and Environment, University of Pisa, Via del Borghetto 80, 56124 Pisa, Italy; 2 CBC (Europe) Srl, Biogard Division, Via Zanica, 25, Grassobbio, BG Italy

**Keywords:** Biodegradable dispenser, IPM, Monitoring, Pyralidae, Phycitinae, Tortricidae

## Abstract

*Lobesia botrana* (Lepidoptera: Tortricidae) and *Cryptoblabes gnidiella* (Lepidoptera: Pyralidae) represent a threat to wine production in Mediterranean countries. In recent years, the development of new formulations promoted the spread of pheromone-based mating disruption (MD) as an effective tool for the management of several insect pests in different agricultural contexts. In this study, we investigated the efficacy of an experimental dispenser designed for simultaneous MD of these two pests. The biodegradable double-tube dispenser (Isonet® L CG-BIOX235) was tested for two years in two Italian wine-growing sites, the first in Apulia (Southern Italy), and the second in Tuscany (Central Italy). Isonet® L CG-BIOX235 efficacy was evaluated by testing different doses (i.e., 300, 400, and 500 dispensers/ha), on different varieties (i.e., Aglianico, Syrah, and Viognier), and comparing it with an untreated control. The MD performed using this dispenser significantly reduced the infestation of both *L. botrana* (i.e., percentage of infested bunches and number of nests per bunch) and *C. gnidiella* compared to the untreated control, although the occurrence of the latter fluctuated throughout the two-year trials. Overall, although our results underline the possibility of combining the pheromones of the two pests in a single dispenser for their simultaneous MD, they also highlight the need for further studies on some aspects of *C. gnidiella* biology and consequently improve the MD efficacy against this species.

**Supplementary Information:**

The online version contains supplementary material available at 10.1007/s11356-024-33980-w.

## Introduction

In most moth species, mating behavior is triggered by a blend of female-emitted sex pheromones (Ando et al. 2004; Greenfield 1981; Jurenka 2003). Perceiving and following these chemical signals, males can locate the calling females. This mechanism led to the evolution of species-specific blends of pheromones that ensure mates to a conspecific signal receiver (Cardé and Haynes 2004; Kennedy et al. 1981). Initially studied just as an intriguing communication system, this chemical and behavioral mechanism is today exploited to manage several harmful crop insects through pheromone-based control strategies, such as sex pheromone-based mating disruption (MD) (Cardé, 1990; Cardé and Minks 1995; Witzgall et al. 2010). Comprising synthetic sex pheromones and other semiochemicals, this technique embodies a promising tool for the control of many insect pests (Mori and Tashiro 2004; Witzgall et al. 2010). The consolidation of MD was driven by the development of a wide range of dispensers, applied at different rates, characterized by a passive or active-release method, made of plastic or biodegradable polymers, and with different amounts of daily released pheromone, according to the target pests behavior and environmental conditions (Benelli et al. 2019; Gordon et al. 2005; Lucchi et al. 2018a; Miller and Gut 2015). Several field tests demonstrated the effectiveness of this technique, disturbing male flight orientation behavior and interfering with mate location in a wide range of agricultural fields (Benelli et al. 2023a; Harari et al. 2007; Ioriatti and Lucchi 2016; Lucchi et al. 2018b).

In the vineyard context, the European Grapevine moth (EGVM) *Lobesia botrana* (Denis and Schiffermüller) (Lepidoptera: Tortricidae) represents one of the most frequent and harmful pest species of grapevine in the Mediterranean basin, as well as in many other countries worldwide (Cooper et al. 2014; Benelli et al. 2023b; Martinez-Sañudo et al. 2013; Reineke and Thiéry 2016; Thiéry et al. 2018). Despite the increasing number of management strategies against EGVM, this is still among the key injurious pests in Central Europe and Mediterranean grape-growing areas. In the latter areas, the EGVM undergoes three generations across the year. First generation larvae (G1) do not usually cause yield losses, as they feed on flowers. On the other hand, the second (G2) and third (G3) generation larvae feed on green and ripening berries respectively and can therefore seriously compromise grape production. This can cause not only a direct decrease in the yield, but also indirect damage caused by an increased susceptibility of the berries to fungal and bacterial infections (Ioriatti et al. 2011; Moschos 2006). The control of this species mainly employs growth regulators and newly developed neurotoxicants (e.g., spinosad) or IPM strategies, including *Bacillus thuringiensis* subsp. *kurstaki* and *aizawaii* (Benelli et al. 2023a; Civolani et al. 2014). The understanding of the courting and mating behavior of EGVM mediated by pheromone blends, now synthesized and formulated, has led to the consolidation and improvement of MD techniques to manage this key pest (Benelli et al. 2023a).

The effectiveness of this technique against EGVM pushed towards the development of new dispensers to manage other grapevine pests, such as the vine mealybug (VMB) *Planococcus ficus* (Signoret) (Hemiptera: Pseudococcidae) (Cocco et al. 2018; Daane et al. 2012; Lucchi et al. 2019a) or the honeydew moth (HM), *Cryptoblabes gnidiella* (Millière) (Lepidoptera, Pyralidae, Phycitinae) (Lucchi et al. 2019b; Ricciardi et al. 2021). Although the latter is a polyphagous species as well, it has been reported to cause severe damage to ripening grapes in warmer coastal vineyards of Central and Southern Italy (Lucchi et al. 2019b). Little is known about its life cycle, but the major damages induced in the vineyards are concurrent with the harvest. This species seems to overwinter both as larvae and pupae under the grapevine bark and, mostly, in dried bunches left in the field after the harvest (Lucchi et al. 2019b). Although HM economic importance was lower in the past due to its poor presence, with the rise of more suitable climatic conditions, HM increased its population in the whole Mediterranean context, including wine-growing areas (Ricciardi et al. 2021). In fact, it can perform from three to four flights during the vine-productive season, with higher peaks starting from the veraison until the harvest (Ricciardi et al. 2021).

To date, HM control is based mainly on insecticide strategies used against EGVM, although they have not always provided satisfactory results (Wysoki et al. 1975). Therefore, MD could be a promising alternative, especially if combined with the same control strategies for the EGVM and other vineyard pests (Harari et al. 2007; Ricciardi et al. 2021; Sellanes and González 2014). With this objective, a newly developed double dispenser for both EGVM and VMB resulted to be an effective tool for the control of both species (Ricciardi et al. 2022).

In this research, we evaluated the effectiveness of the new experimental dispenser Isonet® L CG-BIOX235 (Shin-Etsu Chemical Co. Ltd., Japan); its efficacy for the simultaneous MD of EGVM and HM was assessed over a two-year field trial in two Italian study sites, located in Tuscany (Central Italy) and Apulia (Southern Italy), showing similar pest histories. Three different dispenser rates (300, 400, and 500 dispensers/ha) were tested, and pest populations and crop damage were then compared with an untreated control.

## Materials and methods

### MD dispenser and experimental sites

Isonet® L CG-BIOX235 is a biodegradable dual-capillary tube dispenser containing the synthetic sex pheromone of the two grape key pests EGVM and HM. It consists of two parallel capillary tubes joined and sealed at the ends; one is filled with the EGVM synthetic sex pheromone and the other one with the HM synthetic sex pheromone (Table 1). The device can be easily placed over the end of a spur or looped around cordons thanks to the central slot, which allows each dispenser to form a ring that can be simply deployed.

**Table 1:** Chemical composition of the pheromones contained in the experimental dispenser Isonet® L CG-BIOX235

Experimental dispenser	Target pest	a.s.* content
Isonet® L CG-BIOX235	*Lobesia botrana*	(*E,Z*)-7,9-dodecadienyl acetate (38–51%)
	*Cryptoblabes gnidiella*	(*Z*)-11-hexadecenal (11–15%)
		(*Z*)-13-octadecenal (11–15%)
	Minor component of Lepidoptera pests’ sex pheromone	Tetradecyl acetate (9–14%)

^*^a.s. = active substance

Field trials were conducted in 2022 and 2023 in two typical Italian wine-growing regions with similar pest histories (Table 2) on three different wine grape varieties (Table 3).

**Table 2:** Location of the study vineyards, their pest history, and trial year

Trial	Site	Province	Region	Longitude	Latitude	Pest history (infestation)	Year
1	Castiglione della Pescaia	Grosseto (GR)	Tuscany	10.947019 E	42.810255 N	Medium–high	2022
2	Castiglione della Pescaia	Grosseto (GR)	Tuscany	10.946493 E	42.808499 N	Medium–high	2022
3	Minervino Murge	Barletta-Trani (BT)	Apulia	16.046653 E	41.147319 N	Medium–high	2022
4	Minervino Murge	Barletta-Trani (BT)	Apulia	16.046653 E	41.147319 N	Medium–high	2023

**Table 3:** Details of the vineyards, where Isonet® L CG-BIOX235 dispensers were tested against EGVM and HM

Trial	Crop	Variety	Rootstock	Training system	Plant age (years)
1	Wine grape	Viognier	3309	Guyot	8
2	Wine grape	Syrah	3309	Guyot	18–29
3	Wine grape	Aglianico	420 A	Low cordon	15–18

In 2022, the trials were carried out in Castiglione della Pescaia (Grosseto province, Tuscany) in vineyards of Syrah and Viognier varieties, and in Minervino Murge (Barletta-Trani province, Apulia) in vineyards of Aglianico variety. In 2023, a single MD trial was carried out in the Apulian study site (Table 4). Moreover, both these farms started to apply the MD against the EGVM more than five years before the experiment, but none against the HM, even though its worrying presence. However, depending on year, study site, and farm availability, we evaluated the efficacy of the experimental dispenser Isonet® L CG-BIOX235 compared to an untreated control. The dose–response effect was evaluated by testing this dispenser at 300, 400, and 500 per ha (Table 4).

**Table 4:** Details of the experimental treatments evaluated in two years of study

Study site and year	Treatment 1	Treatment 2	Treatment 3	Untreated Control
Castiglione della Pescaia (Syrah) 2022	-	-	Isonet® L CG-BIOX235 (500 d/ha) (Plot size 3.6 ha)	Yes (Plot size 1,8 ha)
Castiglione della Pescaia (Syrah) 2022	Isonet® L CG-BIOX235 (300 d/ha) (Plot size 1.2 ha)	Isonet® L CG-BIOX235 (400 d/ha) (Plot size 1.2 ha)	Isonet® L CG-BIOX235 (500 d/ha) (Plot size 1.2 ha)	No
Castiglione della Pescaia (Viognier) 2022	-	-	Isonet® L CG-BIOX235 (500 d/ha) (Plot size 2 ha)	Yes (Plot size 0.5 ha)
Minervino Murge (Aglianico) 2022	-	Isonet® L CG-BIOX235 (400 d/ha) (Plot size 5 ha)	Isonet® L CG-BIOX235 (500 d/ha) (Plot size 6.5 ha)	Yes (Plot size 1 ha)
Minervino Murge (Aglianico) 2023	-	Bt (0.75 L/ha) (plot size 1.2 ha)	Isonet® L CG-BIOX235 (500 d/ha) + Bt (0.75 L/ha)(Plot size 5 ha)	Yes (Plot size 0.4 ha)

### Experimental design

According to the criteria ruling MD tests, our trials were carried out on uniform and large plots from 1.2 to 6.5 ha in size. Untreated control plots, ranging from 0.5 to 1.8 ha in size, were smaller than MD plots, but big enough to allow for assessments on the same number of flower clusters and bunches.

All the dispensers were placed in the last decade of March, before the beginning of the first flight of both target pests. For a better evaluation of the effectiveness of the strategy, an untreated control was planned for each year at both study sites. Unfortunately, in 2023, due to production losses related to downy mildew and the risk of further losses associated with the abundant presence of the two pests, insecticide treatments based on *Bacillus thuringiensis* subsp. *kurstaki* (*Bt*) were performed. Treatments were carried out on all the vineyards tested, two against EGVM (12/07 and 20/07) and two against HM (25/08 and 01/09) using Delfin® (0.75 kg/ha). However, it was possible to maintain an untreated control plot until the harvest (Table 4).

### Assessment of EGVM and HM management

The efficacy of Isonet® L CG-BIOX235 was evaluated for both target pests. In each site, plots were divided into subplots, 10 per treatment. Flower clusters and bunches infestation rates were determined for EGVM by sampling 100 flower clusters per sub-plot in the first generation (G1), 100 bunches per sub-plot in the second generation (G2), and 50 bunches per sub-plot in the third generation (G3) resulting in a total of 1000 flower clusters per plot in G1, 1000 bunches in G2 and 500 bunches in G3. Notably, in G1 and G2 surveys were carried out on the flower clusters and green bunches directly on the plant, while in G3 the bunches were collected and inspected ad hoc. Moreover, to provide more reliable data, infestation severity was also assessed by counting the number of nests per flower cluster/bunch in G1, G2, and G3 respectively (reported in ESM).

As regards HM, considering that in G1 and G2 almost no specimens are found on inflorescences or green bunches, samplings were carried out directly at harvest in G3 on 50 bunches per sub-plot, for a total of 500 bunches per plot, assessing the percentage of infested bunches.

To collect more representative data, all the samplings were made on multiple rows simultaneously.

### EGVM and HM males flight monitoring

The flights of EGVM and HM males were monitored through Biogard Delta Traps (BDT) (CBC (Europe) S.r.l., Grassobbio, Italy). EGVM traps were baited with lures containing the main component of the synthetic sex pheromone, (*E*, *Z*)-7,9-dodecadienyl acetate; likewise, HM traps were baited with the main components of its synthetic sex pheromone i.e., (*Z*)-11-hexadecenal (*Z*)-13-octadecenal (CBC (Europe) S.r.l., Grassobbio, Italy). For each plot, two pheromone traps were deployed, one per species, both in untreated control and MD plots. Each trap was checked weekly; the pheromone lures were replaced every four weeks for EGVM and every three weeks for HM given the volatility of the two aldehydes.

### Statistical analysis

Statistical analyses were carried out in R 4.2.1 (R Development Core Team 2008). In each plot’s dataset, we used the “glmmTMB” R package (Brooks et al. 2017) to fit a Generalized Linear Mixed Model and test the efficacy of Isonet® L CG-BIOX235 compared with untreated control, including both species in each model. As predictor variables, we used the percentage of infested bunches in terms of the number of infested bunches per number of sampled bunches, resulting in “1” when infested, and “0” in non-infested ones. As such, we used a Binomial distribution with subplot membership as random effect in each model. We tested model fit using the “DHARMa” package (Hartig 2022) and, next, the “car” package (Fox and Weisberg 2019) to test which factors of the model had a significant effect on the dependent variable. Then, we carried out a post hoc analysis using estimated marginal means with the Bonferroni correction, as implemented by the “emmeans” package (Lenth 2022), to examine the statistical differences between the two groups.

## Results

### Impact on EGVM and HM Infestation

To assess the pest population pressure in each site, EGVM and HM infestation levels were measured as the percentage of infested bunches. In both years, we found significant differences in the infestation levels among the study sites (Tuscany and Apulia in 2022, and Apulia in 2023) for all the three generations of EGVM as well as for HM (for the full statistical analyses, see ESM). In addition, to ensure the efficacy of the experimental product, the EGVM infestation level was recorded as the number of nests per bunch.

#### Tuscany 2022, Syrah variety

**Fig. 1:**
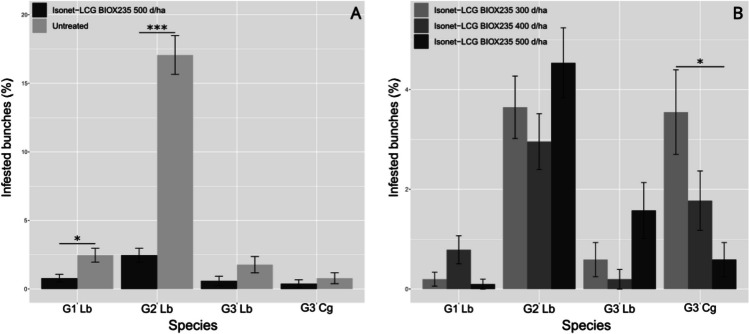
Results of the experimental dispenser tested in 2022 in the Tuscany site of Syrah variety (**A**) and the dose-effects of three different concentrations (**B**). (Asterisk significance code: 0 '***' 0.001 '**' 0.01 '*' 0.05). Lb = *Lobesia botrana*, Cg = *Cryptoblabes gnidiella*

For EGVM, the percentage of infested bunches varies significantly among treatments and for the generation considered. In G1 and G2, the experimental dispenser resulted in significantly lower infestation levels compared with the untreated control (GLMM post hoc, Bonferroni corrected: G1: z = -2.786, *p* = 0.0214; G2: z = -9.043, *p* < 0.0001), while in G3 no statistically significant differences were found among treatments, probably due to decrease of population related to the extreme temperature (~ 40 °C) during the third flight. As for G3, HM seems to be not affected by the experimental dispenser, and analyses showed no effects of any treatment (Fig. 1A; ESM, section 2.1.1).

The number of EGVM nests per bunch reflected the results just reported above: in G1 and G2, the untreated control presented a significantly higher number of nests per bunch (G1: z = -2.598, *p* = 0.281; G2: z = -9.199, *p* < 0.0001), while in G3 there was no difference.

Concerning the dose effect, the only relevant result was about the Isonet® L CG-BIOX235 at 500 dispensers/ha which appeared to be more effective than 300 dispensers/ha, but only for HM (z = 2.908, *p* = 0.0436) (Fig. 1B).

As above, the measured number of nests per bunch did not seem to be affected by the tested doses (ESM, section 2.1.2).

#### Tuscany 2022, Viognier variety

**Fig. 2:**
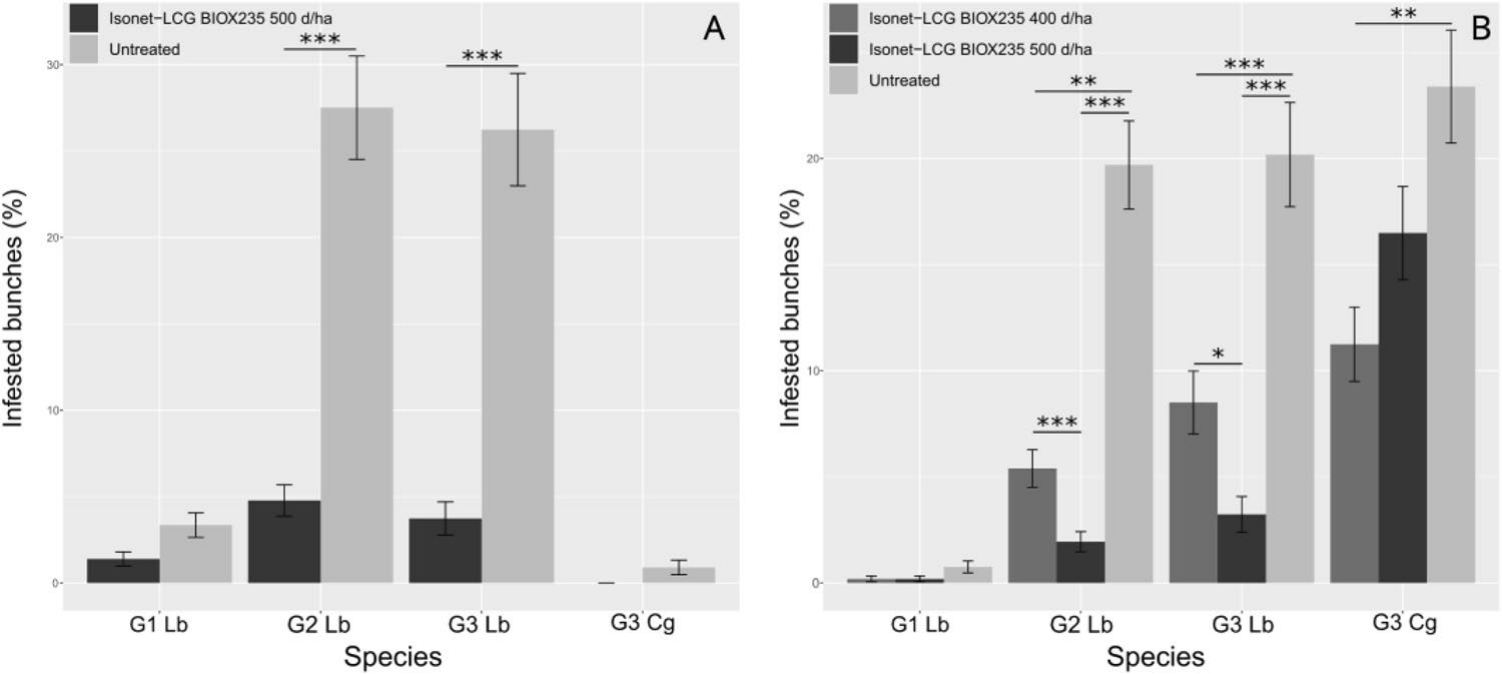
Results of the experimental dispenser tested in 2022 in the Tuscany site of Viognier variety (**A**) and the Apulian site of Aglianico variety (**B**). (Asterisk significance code: 0 '***' 0.001 '**' 0.01 '*' 0.05). Lb = *Lobesia botrana*, Cg = *Cryptoblabes gnidiella*

In G1, the percentage of infested bunches was not significantly higher in control plots (GLMM post hoc, Bonferroni corrected: z = -2.459, *p* = 0.0558), while in the other generations of EGVM emerged a significant effect of Isonet® L CG-BIOX235 with respect to untreated control (G2: z = -8.101, *p* < 0.0001; G3: z = -7.042, *p* < 0.0001). As for the Syrah variety, HM was unaffected by the experimental dispenser (z = -0.008, *p* = 1.0000) (Fig. 2A).

Again, the percentage of bunches infested by EGVM nests almost reflected the aforementioned situation, with a higher number of nests in the untreated control for both G2 and G3 (G2: z = -8.500, *p* < 0.0001; G3: z = -7.166, *p* < 0.0001), but the treatment appeared to influence also the number of nests in G1 (z = -2.687, *p* = 0.0216) (ESM, section 2.1.3).

#### Apulia 2022, Aglianico variety

In EGVM G1, our analysis did not show significant effects among treatments (ESM, section 2.2.1). On the other hand, we found a significant difference between the two doses of the experimental dispenser, with a lower percentage of infested bunches in plots treated with Isonet® L CG-BIOX235 at 500 d/ha than 400 d/ha in G2 (GLMM post hoc, Bonferroni corrected: z = 3.497, *p* = 0.0057) and in G3 (z = 3.109, *p* = 0.0226). However, the two experimental concentrations resulted both effective compared with the untreated control in both G2 and G3 (G2: 400 d/ha: z = -6.715, *p* < 0.0001; 500 d/ha: z = -8.964, *p* < 0.0001; G3: 400 d/ha: z = -4.110, *p* = 0.0005; 500 d/ha: z = -6.547, *p* < 0.0001). For HM, only the 400 d/ha dose resulted in a significant reduction of the infestation compared to the control (z = -3.832, *p* = 0.0015) (Fig. 2B).

Once again, the percentage of infested bunches in terms of EGVM nests retraced the other measurement: in G1 there seemed to be no significant effect of the experimental dispenser (400 d/ha: z = -1.721, *p* = 0.7568; 500 d/ha: z = -1.946, *p* = 0.4650), while in G2 and G3 we found a higher number of nests in untreated subplots than in treated ones (G2: 400 d/ha: z = -6.801, *p* < 0.0001; 500 d/ha: z = -9.006, *p* < 0.0001; G3: 400 d/ha: z = -4.676, *p* < 0.0001; 500 d/ha: z = -7.241, *p* < 0.0001) and the 500 d/ha dose resulted more effective than 400 d/ha in both G2 and G3 (G2: z = 3.507, *p* = 0.0041; G3: z = 3.386; *p* = 0.0064) but not in G1 (z = 0.563, *p* = 1.0000).

#### Apulia 2023, Aglianico variety

In 2023, due to production losses related to the abundant presence of the two pests, insecticide treatments based on *Bacillus thuringiensis* subsp. *kurstaki* (*Bt*) were applied in the tested vineyard. Thus, our statistical models were divided according to the treatment changes.

**Fig. 3:**
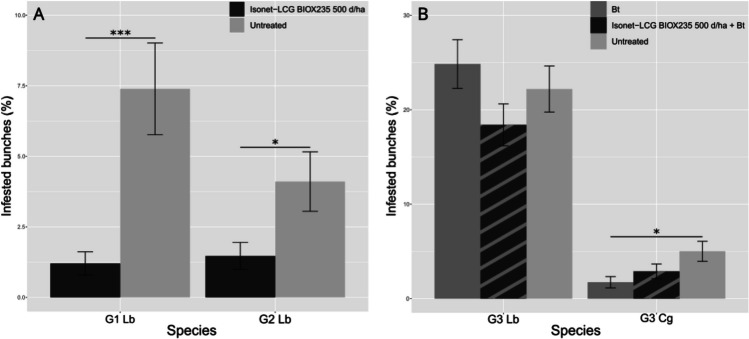
Results of the experimental dispenser tested in 2023 in the Apulian site of Aglianico variety on EGVM G1 and G2 (**A**), and on EGVM G3 and HM (**B**). (Asterisk significance code: 0 '***' 0.001 '**' 0.01 '*' 0.05). Lb = *Lobesia botrana*, Cg = *Cryptoblabes gnidiella*

Both in EGVM G1 and G2, the results showed a significant difference between the experimental dispenser and the untreated control (G1: z = -4.505, *p* < 0.0001; G2: z = -2.550, *p* = 0.0215) (Fig. 3A).

In EGVM and HM G3, the effect of treatment was masked by *Bt*, and no statistical differences between the experimental dispenser and the untreated control emerged (ESM, section 2.2.2) (Fig. 3B).

### Male catches

Our analyses did not find significant differences between the number of males caught weekly in the monitoring traps recorded in MD-treated and untreated vineyards. This was mainly due to the lack of data because the number of males caught by traps was too low. The only relevant results concerned the Apulian site, with the Aglianico variety, in the year 2022. Here, we had enough catches and statistical results showed a significant difference between both Isonet® L CG-BIOX235 at 400 d/ha (z = 3.265, p = 0.0031) and 500 d/ha compared to control (z = 2.344, p = 0.0500), with a higher number of catches in Bt-treated control. However, there is no difference between the two treatments (z = -1.351, p = 0.3672) (ESM, section 2.2.1).

## Discussion

The increasing literature concerning MD strategies and the rising interest in double dispensers for the combined control of different species led to the development of Isonet® L CG-BIOX235. The lack of products to effectively manage HM and the wide use of MD for other species, such as EGVM, make developing a pheromone-based approach for managing this species crucial. According to Ricciardi et al. (2022), to be effective, a double dispenser must release an adequate amount of both pheromones, and the chemical signal must cover the entire period of mating activity of both pests until grape harvest. Furthermore, the MD formulations should be effective in cases of high pest population densities under different climatic conditions (Ioriatti et al. 2011; Lucchi et al. 2019b). For EGVM, previous studies reported the effectiveness of MD strategies against this species, with different kind of dispensers (e.g., ampulla and capillary tube) and pheromone release modalities (e.g., active or passive release devices) (Altindisli et al. 2016; Benelli et al. 2023c; Gordon et al. 2005; Ioriatti et al. 2011; Lucchi et al. 2018a). In our trials, the experimental dispenser proved to be effective at all densities tested, showing a comparable reduction in EGVM infestation levels. The exception is reported in the Apulian study site, where two different densities were tested. Here, the presence of EGVM was lower in plots treated with a dose of 500 d/ha than in those with 400 d/ha, although they were both effective in reducing the infestation for G2 and G3 compared with the untreated control. Although the use of a different number of dispensers per hectare could result in varying effectiveness of MD (e.g., Epstein et al. 2006), in our study the two densities/ha tested in Apulia on the Aglianico variety and the three densities/ha tested in Tuscany on the Syrah variety resulted effective in reducing the infestation level, at least as far as EGVM is concerned. Conversely, for HM no clear dose response effect emerged: in plots of Aglianico with a lower concentration of dispensers per hectare (400 d/ha) we found a reduced presence of HM than in plots treated with a higher dose (500 d/ha). On the other hand, our results show that a high dispenser density per hectare, such as the 500 d/ha dose reported in Tuscany for the Syrah variety, could be a better alternative to the lower dose of 300 d/ha. Similar results were obtained by Ricciardi et al. (2021), where the tested MD dispenser was effective on this species depending on year and study site. However, these puzzling results may be due to the low occurrence of this species in the considered study sites. Due to the almost absent male catches in the pheromone traps (Atanassov et al. 2002; Miller and Gut 2015) and the low percentage of infestation throughout the season, it was not possible to obtain enough reliable data to assess the effectiveness of Isonet® L CG-BIOX235 MD on HM.

However, before stating the effectiveness of an MD strategy against this species, we should investigate all the variables (at first some aspects of its biology that are not yet very clear, such as oviposition sites during the first and second flight) involved in the development of this strategy. Previous studies suggest that the use of synthetic pheromones can reduce HM damage, but the understanding of the application of these compounds in control strategies is poorly unraveled and their use still lacks further experimentation (Acín, 2019; Sellanes and González 2014). The development of a double dispenser to manage various species simultaneously may represent an effective and sustainable tool for the control of vineyard pest species especially where winegrowers must manage several harmful insect species in the same vineyard. Furthermore, while one of the limitations of the MD for single-species management could be the high production cost of synthetic pheromones and dispensers, the option of combining the pheromone of several species in a single device could represent a simplification in insect management and at the same time an economic advantage for the stakeholders (Benelli et al. 2019; Lucchi and Benelli 2018). Some evidence of the possibility of exploiting two pheromones in a single dispenser for the management of multiple pests began years ago (Hull et al. 2009; Il’ichev et al. 2007; Ioriatti et al. 2004, 2008; Mitchell et al. 1997; Ricciardi et al. 2022). Notably, the analysis carried out by Il'ichev et al. (2007) showed that managing *Cydia pomonella* L. (Lepidoptera: Tortricidae) and *Grapholita molesta* (Busck) (Lepidoptera: Tortricidae) with a double dispenser reduced the cost by half compared to that which would have been sustained by employing single dispensers for each species. Additional benefits could result from using biodegradable dispensers, which would lower the costs of their removal and disposal. In this perspective, our experimental dispenser is made of biodegradable material, thus impacting less on the environment than those of plastic materials (Lucchi et al. 2018a; Ricciardi et al. 2022). This could be a good starting point in the development of a new double dispenser successful against HM and other vineyard pest species. However, results of this study underline that it is possible to combine the pheromone of these two species for simultaneous MD of both, but at the same time reveals some limitations of MD against HM. Indeed, as already emerged in related studies (Ricciardi et al. 2021), further investigations are needed to validate and improve its efficacy.

### Supplementary Information

Below is the link to the electronic supplementary material.

Supplementary file1 (HTML 1870 KB)

## Data Availability

The data that support the findings of this study are available from the corresponding author upon reasonable request.
