# Supplementary material for: Simultaneous mating disruption of two moth pests of the vineyard (Lobesia botrana and Cryptoblabes gnidiella) through a biodegradable sex pheromone dispenser
Source: Environ Sci Pollut Res Int. 2024 Jun 24;31(31):43865–73. doi: 10.1007/s11356-024-33980-w (PMC11252198; doi:10.1007/s11356-024-33980-w)
Supplement: Supplementary file 1 — Supplementary file1 (HTML 1870 KB) [file 11356_2024_33980_MOESM1_ESM.html]

ESM - Analysis | Simultaneous mating disruption of two moth pests of the vineyard (Lobesia botrana and Cryptoblabes gnidiella) through a biodegradable sex pheromone dispenser


Code 

- Show All Code
- Hide All Code

# ESM - Analysis | Simultaneous mating disruption of two moth pests of the vineyard (*Lobesia botrana* and *Cryptoblabes gnidiella*) through a biodegradable sex pheromone dispenser

## ESM - Analysis | Simultaneous mating disruption of two moth pests of the vineyard (*Lobesia botrana* and *Cryptoblabes gnidiella*) through a biodegradable sex pheromone dispenser

- 1 Setup
  - 1.1 Prepare R
    environment
- 2 Analyses
  - 2.1 Tuscany
    - 2.1.1 Syrah 2022
    - 2.1.2 Syrah 2022 - Dose
      effect
    - 2.1.3 Viognier 2022
  - 2.2 Apulia
    - 2.2.1 Aglianico 2022
    - 2.2.2 Aglianico 2023
- 3 Session info

2024-02-21

## Renato Ricciardi¹, Livia De Fazi¹, Giordana D’Anna¹, Francesco Savino², Edith Ladurner², Andrea Iodice², Giovanni Benelli¹, Andrea Lucchi¹

#### ¹ Department of Agriculture, Food and Environment, University of Pisa, via del Borghetto 80, 56124 Pisa, Italy

#### ² CBC (Europe) srl, Biogard Division, Via Zanica, 25, Grassobbio (BG), Italy

### Corresponding Author: giovanni.benelli@unipi.it

---

This document (ESM) contains the statistical analysis output for the
manuscript. Note that much of the analysis is divided into sections
using tabs. Click on the section you wish to inspect, and scroll down
for the full analysis.

---

# 1 Setup

## 1.1 Prepare R environment

```
library(reshape2)
library(glmmTMB)
library(DHARMa)
library(car)
library(emmeans) 
library(ggplot2) 
library(ggpattern)
library(knitr)
```

# 2 Analyses

## 2.1 Tuscany

### 2.1.1 Syrah 2022

**Load data**

```
inputS <- read.csv2(("analysis_syrah.csv"))
inputS <- subset(inputS,Treatment!='Isonet L TT 250d/ha + CRYX2021 400 d/ha')
kable(head(inputS))
```

| Treatment | rep | N..sampled.bunches | N..infested.bunches | P\_inf | Gen | Sp | n.nests.bunch |
| --- | --- | --- | --- | --- | --- | --- | --- |
| Untreated | 1 | 50 | 3 | 6 | G3 Lb | Lb | 0.06 |
| Untreated | 2 | 50 | 0 | 0 | G3 Lb | Lb | 0.00 |
| Untreated | 3 | 50 | 1 | 2 | G3 Lb | Lb | 0.02 |
| Untreated | 4 | 50 | 2 | 4 | G3 Lb | Lb | 0.04 |
| Untreated | 5 | 50 | 0 | 0 | G3 Lb | Lb | 0.00 |
| Untreated | 6 | 50 | 1 | 2 | G3 Lb | Lb | 0.02 |

reshaping data

```
tab0 <- data.frame(Treatment=character(),
                 Sb=integer(), 
                 Gen=character(), 
                 Species=character(),
                 Binary=integer(),
                 stringsAsFactors=FALSE)

for (i in 1:length(inputS$Treatment)){
  n.bunches <- inputS$N..sampled.bunches[i]
  Treatment <- rep(inputS$Treatment[i], n.bunches)
  Sb <- rep(inputS$rep[i], n.bunches)
  Binary <- c(rep(1, inputS$N..infested.bunches[i]), rep(0, n.bunches - inputS$N..infested.bunches[i]))
  Gen <- rep(inputS$Gen[i], n.bunches)
  if(Gen[1]=="G3 zCg"){Species <- rep("Cg", n.bunches)} else {Species <- rep("Lb", n.bunches)}
  toadd <- data.frame(Treatment, Sb, Gen, Species, Binary)
  tab0 <- rbind(tab0, toadd)
}

tab0$onlygen <- tab0$Gen
tab0$onlygen[tab0$onlygen == "G3 Lb"] <-"G3"
tab0$onlygen[tab0$onlygen == "G3 zCg"] <-"G3"
tab0$onlygen[tab0$onlygen == "G1 Lb"] <-"G1"
tab0$onlygen[tab0$onlygen == "G2 Lb"] <-"G2"

tab0$rand <- paste("plot", tab0$Sb, tab0$Treatment, tab0$onlygen)

tab0$rand <- as.factor(tab0$rand)
tab0$Treatment <- as.factor(tab0$Treatment)
```

**Model**

```
model0 <- glmmTMB(Binary ~Treatment*Gen + (1|rand), data=tab0, family=binomial())
```

```
Anova(model0)
```

```
## Analysis of Deviance Table (Type II Wald chisquare tests)
## 
## Response: Binary
##                  Chisq Df Pr(>Chisq)    
## Treatment      86.2792  1    < 2e-16 ***
## Gen           151.8581  3    < 2e-16 ***
## Treatment:Gen   6.5877  3    0.08627 .  
## ---
## Signif. codes:  0 '***' 0.001 '**' 0.01 '*' 0.05 '.' 0.1 ' ' 1
```

```
e <- emmeans(model0, ~Treatment*Gen, type='response')
e
```

```
##  Treatment                   Gen       prob      SE  df asymp.LCL asymp.UCL
##  Isonet-LCG BIOX235 500 d/ha G1 Lb  0.00789 0.00281 Inf  0.003918    0.0158
##  Untreated                   G1 Lb  0.02470 0.00506 Inf  0.016500    0.0368
##  Isonet-LCG BIOX235 500 d/ha G2 Lb  0.02469 0.00506 Inf  0.016497    0.0368
##  Untreated                   G2 Lb  0.17065 0.01414 Inf  0.144700    0.2002
##  Isonet-LCG BIOX235 500 d/ha G3 Lb  0.00592 0.00342 Inf  0.001900    0.0183
##  Untreated                   G3 Lb  0.01776 0.00595 Inf  0.009184    0.0341
##  Isonet-LCG BIOX235 500 d/ha G3 zCg 0.00395 0.00279 Inf  0.000983    0.0157
##  Untreated                   G3 zCg 0.00789 0.00396 Inf  0.002947    0.0210
## 
## Confidence level used: 0.95 
## Intervals are back-transformed from the logit scale
```

```
test(pairs(e, simple = "Treatment"), by = NULL, adjust = "bonferroni")
```

```
##  contrast                                  Gen    odds.ratio     SE  df null
##  (Isonet-LCG BIOX235 500 d/ha) / Untreated G1 Lb       0.314 0.1306 Inf    1
##  (Isonet-LCG BIOX235 500 d/ha) / Untreated G2 Lb       0.123 0.0285 Inf    1
##  (Isonet-LCG BIOX235 500 d/ha) / Untreated G3 Lb       0.329 0.2219 Inf    1
##  (Isonet-LCG BIOX235 500 d/ha) / Untreated G3 zCg      0.498 0.4341 Inf    1
##  z.ratio p.value
##   -2.786  0.0214
##   -9.043  <.0001
##   -1.648  0.3971
##   -0.800  1.0000
## 
## P value adjustment: bonferroni method for 4 tests 
## Tests are performed on the log odds ratio scale
```

```
toplot <- as.data.frame(e)


ggplot(toplot, aes(x=Gen, y=prob*100, fill=Treatment))+
geom_bar(stat="identity", position=position_dodge())+
  geom_errorbar(aes(ymin=(prob-SE)*100, ymax=(prob+SE)*100), position = position_dodge(.9), width=0.2)+
  labs(title = "Tuscany 2022 (Syrah)", x= 'Species', y='Infested bunches (%)')+
  theme(plot.title = element_text(hjust = 0.5))+
scale_fill_manual(values = c('#333333', '#BDBEBE'))+
  scale_x_discrete(labels=c('G1 Lb', 'G2 Lb', 'G3 Lb', 'G3 Cg'))
```

**Number of EGVM nests per bunches**

```
inputS <- na.omit(inputS)
inputS$n.nests.bunch <- (inputS$n.nests.bunch)*(inputS$N..sampled.bunches)
```

```
tab0 <- data.frame(Treatment=character(),
                 Sb=integer(), 
                 Gen=character(),
                 Bin=integer(),
                 stringsAsFactors=FALSE)

for (i in 1:length(inputS$Treatment)){
  n.bunches <- inputS$N..sampled.bunches[i]
  Treatment <- rep(inputS$Treatment[i], n.bunches)
  Sb <- rep(inputS$rep[i], n.bunches)
  Gen <- rep(inputS$Gen[i], n.bunches)
  Bin <- c(rep(1, inputS$n.nests.bunch[i]), rep(0, n.bunches - inputS$n.nests.bunch[i]))
  if(Gen[1]=="G3 zCg"){Species <- rep("Cg", n.bunches)} else {Species <- rep("Lb", n.bunches)}
  toadd <- data.frame(Treatment, Sb, Gen, Bin)
  tab0 <- rbind(tab0, toadd)
}

tab0$rand <- paste("plot", tab0$Sb, tab0$Treatment, tab0$Gen)

tab0$rand <- as.factor(tab0$rand)
tab0$Treatment <- as.factor(tab0$Treatment)
```

**Model**

```
modelN0 <- glmmTMB(Bin ~Treatment*Gen + (1|rand), data=tab0, family=binomial())
```

```
Anova(modelN0)
```

```
## Analysis of Deviance Table (Type II Wald chisquare tests)
## 
## Response: Bin
##                  Chisq Df Pr(>Chisq)    
## Treatment      87.1601  1    < 2e-16 ***
## Gen           122.8621  2    < 2e-16 ***
## Treatment:Gen   6.9123  2    0.03155 *  
## ---
## Signif. codes:  0 '***' 0.001 '**' 0.01 '*' 0.05 '.' 0.1 ' ' 1
```

```
e <- emmeans(modelN0, ~Treatment*Gen, type='response')
e
```

```
##  Treatment                   Gen      prob      SE  df asymp.LCL asymp.UCL
##  Isonet-LCG BIOX235 500 d/ha G1 Lb 0.00885 0.00299 Inf   0.00456    0.0171
##  Untreated                   G1 Lb 0.02463 0.00508 Inf   0.01641    0.0368
##  Isonet-LCG BIOX235 500 d/ha G2 Lb 0.02463 0.00508 Inf   0.01641    0.0368
##  Untreated                   G2 Lb 0.17831 0.01490 Inf   0.15095    0.2094
##  Isonet-LCG BIOX235 500 d/ha G3 Lb 0.00590 0.00342 Inf   0.00189    0.0183
##  Untreated                   G3 Lb 0.01772 0.00595 Inf   0.00914    0.0341
## 
## Confidence level used: 0.95 
## Intervals are back-transformed from the logit scale
```

```
test(pairs(e, simple = "Treatment"), by = NULL, adjust = "bonferroni")
```

```
##  contrast                                  Gen   odds.ratio     SE  df null
##  (Isonet-LCG BIOX235 500 d/ha) / Untreated G1 Lb      0.354 0.1415 Inf    1
##  (Isonet-LCG BIOX235 500 d/ha) / Untreated G2 Lb      0.116 0.0272 Inf    1
##  (Isonet-LCG BIOX235 500 d/ha) / Untreated G3 Lb      0.329 0.2221 Inf    1
##  z.ratio p.value
##   -2.598  0.0281
##   -9.199  <.0001
##   -1.647  0.2990
## 
## P value adjustment: bonferroni method for 3 tests 
## Tests are performed on the log odds ratio scale
```

```
toplot <- as.data.frame(e)


ggplot(toplot, aes(x=Gen, y=prob*100, fill=Treatment))+
geom_bar(stat="identity", position=position_dodge())+
  geom_errorbar(aes(ymin=(prob-SE)*100, ymax=(prob+SE)*100), position = position_dodge(.9), width=0.2)+
  labs(title = "Tuscany 2022 (Syrah)", x= 'Species', y='Nest per bunches (%)')+
  theme(plot.title = element_text(hjust = 0.5))+
scale_fill_manual(values = c('#333333', '#BDBEBE'))+
  scale_x_discrete(labels=c('G1 Lb', 'G2 Lb', 'G3 Lb'))
```

### 2.1.2 Syrah 2022 - Dose effect

**Load data**

```
inputD <- read.csv2(("analysis_syrah_dose.csv"))
kable(head(inputD))
```

| Treatment | Subplot | N..sampled.bunches | N..infested.bunches | P\_inf | Gen | n.nests.bunch |
| --- | --- | --- | --- | --- | --- | --- |
| Isonet-LCG BIOX235 300 d/ha | 1 | 50 | 5 | 10 | G3 zCg | NA |
| Isonet-LCG BIOX235 300 d/ha | 2 | 50 | 0 | 0 | G3 zCg | NA |
| Isonet-LCG BIOX235 300 d/ha | 3 | 50 | 0 | 0 | G3 zCg | NA |
| Isonet-LCG BIOX235 300 d/ha | 4 | 50 | 1 | 2 | G3 zCg | NA |
| Isonet-LCG BIOX235 300 d/ha | 5 | 50 | 2 | 4 | G3 zCg | NA |
| Isonet-LCG BIOX235 300 d/ha | 6 | 50 | 0 | 0 | G3 zCg | NA |

```
tab1 <- data.frame(Treatment=character(),
                 Sb=integer(), 
                 Gen=character(), 
                 Species=character(),
                 Binary=integer(),
                 stringsAsFactors=FALSE)

for (i in 1:length(inputD$Treatment)){
  n.bunches <- inputD$N..sampled.bunches[i]
  Treatment <- rep(inputD$Treatment[i], n.bunches)
  Sb <- rep(inputD$Subplot[i], n.bunches)
  Binary <- c(rep(1, inputD$N..infested.bunches[i]), rep(0, n.bunches - inputD$N..infested.bunches[i]))
  Gen <- rep(inputD$Gen[i], n.bunches)
  if(Gen[1]=="G3 zCg"){Species <- rep("Cg", n.bunches)} else {Species <- rep("Lb", n.bunches)}
  toadd <- data.frame(Treatment, Sb, Gen, Species, Binary)
  tab1 <- rbind(tab1, toadd)
}

tab1$onlygen <- tab1$Gen
tab1$onlygen[tab1$onlygen == "G3 Lb"] <-"G3"
tab1$onlygen[tab1$onlygen == "G3 zCg"] <-"G3"
tab1$onlygen[tab1$onlygen == "G1 Lb"] <-"G1"
tab1$onlygen[tab1$onlygen == "G2 Lb"] <-"G2"

tab1$rand <- paste("plot", tab1$Sb, tab1$Treatment, tab1$onlygen)

tab1$rand <- as.factor(tab1$rand)
tab1$Treatment <- as.factor(tab1$Treatment)
```

**Model**

```
model1 <- glmmTMB(Binary ~Treatment*Gen + (1|Sb), data=tab1, family=binomial())
```

```
Anova(model1)
```

```
## Analysis of Deviance Table (Type II Wald chisquare tests)
## 
## Response: Binary
##                 Chisq Df Pr(>Chisq)    
## Treatment      2.3719  2   0.305455    
## Gen           51.4698  3  3.885e-11 ***
## Treatment:Gen 22.0296  6   0.001196 ** 
## ---
## Signif. codes:  0 '***' 0.001 '**' 0.01 '*' 0.05 '.' 0.1 ' ' 1
```

```
e <- emmeans(model1, ~Treatment*Gen, type='response')
e
```

```
##  Treatment                   Gen        prob       SE  df asymp.LCL asymp.UCL
##  Isonet-LCG BIOX235 300 d/ha G1 Lb  0.001967 0.001395 Inf  0.000490   0.00787
##  Isonet-LCG BIOX235 400 d/ha G1 Lb  0.007871 0.002813 Inf  0.003901   0.01582
##  Isonet-LCG BIOX235 500 d/ha G1 Lb  0.000984 0.000985 Inf  0.000138   0.00697
##  Isonet-LCG BIOX235 300 d/ha G2 Lb  0.036455 0.006261 Inf  0.025986   0.05092
##  Isonet-LCG BIOX235 400 d/ha G2 Lb  0.029549 0.005596 Inf  0.020349   0.04273
##  Isonet-LCG BIOX235 500 d/ha G2 Lb  0.045342 0.007044 Inf  0.033374   0.06133
##  Isonet-LCG BIOX235 300 d/ha G3 Lb  0.005903 0.003417 Inf  0.001893   0.01825
##  Isonet-LCG BIOX235 400 d/ha G3 Lb  0.001967 0.001969 Inf  0.000276   0.01388
##  Isonet-LCG BIOX235 500 d/ha G3 Lb  0.015749 0.005606 Inf  0.007814   0.03148
##  Isonet-LCG BIOX235 300 d/ha G3 zCg 0.035468 0.008475 Inf  0.022128   0.05639
##  Isonet-LCG BIOX235 400 d/ha G3 zCg 0.017719 0.005951 Inf  0.009145   0.03406
##  Isonet-LCG BIOX235 500 d/ha G3 zCg 0.005903 0.003417 Inf  0.001893   0.01825
## 
## Confidence level used: 0.95 
## Intervals are back-transformed from the logit scale
```

```
test(pairs(e, simple = "Treatment"), by = NULL, adjust = "bonferroni")
```

```
##  contrast                                                      Gen   
##  (Isonet-LCG BIOX235 300 d/ha) / (Isonet-LCG BIOX235 400 d/ha) G1 Lb 
##  (Isonet-LCG BIOX235 300 d/ha) / (Isonet-LCG BIOX235 500 d/ha) G1 Lb 
##  (Isonet-LCG BIOX235 400 d/ha) / (Isonet-LCG BIOX235 500 d/ha) G1 Lb 
##  (Isonet-LCG BIOX235 300 d/ha) / (Isonet-LCG BIOX235 400 d/ha) G2 Lb 
##  (Isonet-LCG BIOX235 300 d/ha) / (Isonet-LCG BIOX235 500 d/ha) G2 Lb 
##  (Isonet-LCG BIOX235 400 d/ha) / (Isonet-LCG BIOX235 500 d/ha) G2 Lb 
##  (Isonet-LCG BIOX235 300 d/ha) / (Isonet-LCG BIOX235 400 d/ha) G3 Lb 
##  (Isonet-LCG BIOX235 300 d/ha) / (Isonet-LCG BIOX235 500 d/ha) G3 Lb 
##  (Isonet-LCG BIOX235 400 d/ha) / (Isonet-LCG BIOX235 500 d/ha) G3 Lb 
##  (Isonet-LCG BIOX235 300 d/ha) / (Isonet-LCG BIOX235 400 d/ha) G3 zCg
##  (Isonet-LCG BIOX235 300 d/ha) / (Isonet-LCG BIOX235 500 d/ha) G3 zCg
##  (Isonet-LCG BIOX235 400 d/ha) / (Isonet-LCG BIOX235 500 d/ha) G3 zCg
##  odds.ratio    SE  df null z.ratio p.value
##       0.248 0.197 Inf    1  -1.758  0.9440
##       2.002 2.454 Inf    1   0.566  1.0000
##       8.058 8.554 Inf    1   1.966  0.5922
##       1.243 0.311 Inf    1   0.869  1.0000
##       0.797 0.180 Inf    1  -1.008  1.0000
##       0.641 0.153 Inf    1  -1.859  0.7568
##       3.012 3.484 Inf    1   0.954  1.0000
##       0.371 0.252 Inf    1  -1.458  1.0000
##       0.123 0.131 Inf    1  -1.971  0.5852
##       2.039 0.843 Inf    1   1.723  1.0000
##       6.193 3.883 Inf    1   2.908  0.0436
##       3.038 2.035 Inf    1   1.659  1.0000
## 
## P value adjustment: bonferroni method for 12 tests 
## Tests are performed on the log odds ratio scale
```

**Plot**

```
toplot <- as.data.frame(e)

ggplot(toplot, aes(x=Gen, y=prob*100, fill=Treatment))+
  geom_bar(stat="identity", position=position_dodge())+
  geom_errorbar(aes(ymin=(prob-SE)*100, ymax=(prob+SE)*100), position = position_dodge(.9), width=0.2)+
  labs(title = "Tuscany 2022 (Syrah) - Dose effect", x= 'Species', y='Infested bunches (%)')+
  theme(plot.title = element_text(hjust = 0.5))+
scale_fill_manual(values = c('#9E9E9E', '#6E6E6E', '#333333'))+
  scale_x_discrete(labels=c('G1 Lb', 'G2 Lb', 'G3 Lb', 'G3 Cg'))+
  ylim(NA,5.5)
```

**Number of nest per bunches**

```
inputD <- na.omit(inputD)

inputD$n.nests.bunch <- (inputD$n.nests.bunch)*(inputD$N..sampled.bunches)

tab1 <- data.frame(Treatment=character(),
                 Sb=integer(), 
                 Gen=character(),
                 Bin=integer(),
                 stringsAsFactors=FALSE)

for (i in 1:length(inputD$Treatment)){
  n.bunches <- inputD$N..sampled.bunches[i]
  Treatment <- rep(inputD$Treatment[i], n.bunches)
  Sb <- rep(inputD$Subplot[i], n.bunches)
  Gen <- rep(inputD$Gen[i], n.bunches)
  Bin <- c(rep(1, inputD$n.nests.bunch[i]), rep(0, n.bunches - inputD$n.nests.bunch[i]))
  toadd <- data.frame(Treatment, Sb, Gen, Bin)
  tab1 <- rbind(tab1, toadd)
}

tab1$rand <- paste("plot", tab1$Sb, tab1$Treatment, tab1$Gen)

tab1$rand <- as.factor(tab1$rand)
tab1$Treatment <- as.factor(tab1$Treatment)
```

**Model**

```
modelN1 <- glmmTMB(Bin ~Treatment*Gen + (1|rand), data=tab1, family=binomial())
```

```
Anova(modelN1)
```

```
## Analysis of Deviance Table (Type II Wald chisquare tests)
## 
## Response: Bin
##                Chisq Df Pr(>Chisq)    
## Treatment      2.761  2    0.25145    
## Gen           47.645  2  4.508e-11 ***
## Treatment:Gen 10.664  4    0.03061 *  
## ---
## Signif. codes:  0 '***' 0.001 '**' 0.01 '*' 0.05 '.' 0.1 ' ' 1
```

```
e <- emmeans(modelN1, ~Treatment*Gen, type='response')
e
```

```
##  Treatment                   Gen       prob       SE  df asymp.LCL asymp.UCL
##  Isonet-LCG BIOX235 300 d/ha G1 Lb 0.001903 0.001362 Inf  0.000468   0.00772
##  Isonet-LCG BIOX235 400 d/ha G1 Lb 0.007619 0.002820 Inf  0.003682   0.01570
##  Isonet-LCG BIOX235 500 d/ha G1 Lb 0.000951 0.000957 Inf  0.000132   0.00681
##  Isonet-LCG BIOX235 300 d/ha G2 Lb 0.035218 0.006912 Inf  0.023914   0.05158
##  Isonet-LCG BIOX235 400 d/ha G2 Lb 0.028683 0.006038 Inf  0.018945   0.04321
##  Isonet-LCG BIOX235 500 d/ha G2 Lb 0.044190 0.007875 Inf  0.031083   0.06247
##  Isonet-LCG BIOX235 300 d/ha G3 Lb 0.005714 0.003354 Inf  0.001804   0.01795
##  Isonet-LCG BIOX235 400 d/ha G3 Lb 0.001903 0.001914 Inf  0.000265   0.01355
##  Isonet-LCG BIOX235 500 d/ha G3 Lb 0.015214 0.005622 Inf  0.007351   0.03123
## 
## Confidence level used: 0.95 
## Intervals are back-transformed from the logit scale
```

```
test(pairs(e, simple = "Treatment"), by = NULL, adjust = "bonferroni")
```

```
##  contrast                                                      Gen   odds.ratio
##  (Isonet-LCG BIOX235 300 d/ha) / (Isonet-LCG BIOX235 400 d/ha) G1 Lb      0.248
##  (Isonet-LCG BIOX235 300 d/ha) / (Isonet-LCG BIOX235 500 d/ha) G1 Lb      2.002
##  (Isonet-LCG BIOX235 400 d/ha) / (Isonet-LCG BIOX235 500 d/ha) G1 Lb      8.061
##  (Isonet-LCG BIOX235 300 d/ha) / (Isonet-LCG BIOX235 400 d/ha) G2 Lb      1.236
##  (Isonet-LCG BIOX235 300 d/ha) / (Isonet-LCG BIOX235 500 d/ha) G2 Lb      0.790
##  (Isonet-LCG BIOX235 400 d/ha) / (Isonet-LCG BIOX235 500 d/ha) G2 Lb      0.639
##  (Isonet-LCG BIOX235 300 d/ha) / (Isonet-LCG BIOX235 400 d/ha) G3 Lb      3.014
##  (Isonet-LCG BIOX235 300 d/ha) / (Isonet-LCG BIOX235 500 d/ha) G3 Lb      0.372
##  (Isonet-LCG BIOX235 400 d/ha) / (Isonet-LCG BIOX235 500 d/ha) G3 Lb      0.123
##     SE  df null z.ratio p.value
##  0.200 Inf    1  -1.729  0.7537
##  2.472 Inf    1   0.563  1.0000
##  8.639 Inf    1   1.947  0.4634
##  0.358 Inf    1   0.731  1.0000
##  0.213 Inf    1  -0.878  1.0000
##  0.179 Inf    1  -1.599  0.9877
##  3.514 Inf    1   0.946  1.0000
##  0.259 Inf    1  -1.421  1.0000
##  0.132 Inf    1  -1.950  0.4604
## 
## P value adjustment: bonferroni method for 9 tests 
## Tests are performed on the log odds ratio scale
```

```
toplot <- as.data.frame(e)


ggplot(toplot, aes(x=Gen, y=prob*100, fill=Treatment))+
geom_bar(stat="identity", position=position_dodge())+
  geom_errorbar(aes(ymin=(prob-SE)*100, ymax=(prob+SE)*100), position = position_dodge(.9), width=0.2)+
  labs(title = "Tuscany 2022 (Syrah) - Dose effect", x= 'Species', y='Nest per bunches (%)')+
  theme(plot.title = element_text(hjust = 0.5))+
scale_fill_manual(values = c('#9E9E9E', '#6E6E6E', '#333333'))+
  scale_x_discrete(labels=c('G1 Lb', 'G2 Lb', 'G3 Lb'))
```

### 2.1.3 Viognier 2022

**Load data**

```
inputV <- read.csv2(("analysis_viognier.csv"))
inputV <- subset(inputV,Treatment!='Isonet L TT 250d/ha + CRYX2021 400 d/ha')
kable(head(inputV))
```

| Treatment | rep | N..sampled.bunches | N..infested.bunches | P\_inf | Gen | n.nests.bunch |
| --- | --- | --- | --- | --- | --- | --- |
| Untreated | 1 | 100 | 6 | 6 | G1 Lb | 0.06 |
| Untreated | 2 | 100 | 4 | 4 | G1 Lb | 0.06 |
| Untreated | 3 | 100 | 5 | 5 | G1 Lb | 0.05 |
| Untreated | 4 | 100 | 7 | 7 | G1 Lb | 0.08 |
| Untreated | 5 | 100 | 1 | 1 | G1 Lb | 0.01 |
| Untreated | 6 | 100 | 5 | 5 | G1 Lb | 0.06 |

```
tab2 <- data.frame(Treatment=character(),
                 Sb=integer(), 
                 Gen=character(), 
                 Species=character(),
                 Binary=integer(),
                 stringsAsFactors=FALSE)

for (i in 1:length(inputV$Treatment)){
  n.bunches <- inputV$N..sampled.bunches[i]
  Treatment <- rep(inputV$Treatment[i], n.bunches)
  Sb <- rep(inputV$rep[i], n.bunches)
  Binary <- c(rep(1, inputV$N..infested.bunches[i]), rep(0, n.bunches - inputV$N..infested.bunches[i]))
  Gen <- rep(inputV$Gen[i], n.bunches)
  if(Gen[1]=="G3 zCg"){Species <- rep("Cg", n.bunches)} else {Species <- rep("Lb", n.bunches)}
  toadd <- data.frame(Treatment, Sb, Gen, Species, Binary)
  tab2 <- rbind(tab2, toadd)
}

tab2$onlygen <- tab2$Gen
tab2$onlygen[tab2$onlygen == "G3 Lb"] <-"G3"
tab2$onlygen[tab2$onlygen == "G3 zCg"] <-"G3"
tab2$onlygen[tab2$onlygen == "G1 Lb"] <-"G1"
tab2$onlygen[tab2$onlygen == "G2 Lb"] <-"G2"

tab2$rand <- paste("plot", tab2$Sb, tab2$Treatment, tab2$onlygen)

tab2$rand <- as.factor(tab2$rand)
tab2$Treatment <- as.factor(tab2$Treatment)
```

**Model**

```
model2 <- glmmTMB(Binary ~Treatment*Gen + (1|rand), data=tab2, family=binomial())
```

```
Anova(model2)
```

```
## Analysis of Deviance Table (Type II Wald chisquare tests)
## 
## Response: Binary
##                  Chisq Df Pr(>Chisq)    
## Treatment     112.1620  1    < 2e-16 ***
## Gen           155.4830  3    < 2e-16 ***
## Treatment:Gen   8.5932  3    0.03522 *  
## ---
## Signif. codes:  0 '***' 0.001 '**' 0.01 '*' 0.05 '.' 0.1 ' ' 1
```

```
e <- emmeans(model2, ~Treatment*Gen, type='response')
e
```

```
##  Treatment                   Gen      prob       SE  df asymp.LCL asymp.UCL
##  Isonet-LCG BIOX235 500 d/ha G1 Lb  0.0139 4.05e-03 Inf   0.00786    0.0245
##  Untreated                   G1 Lb  0.0335 7.08e-03 Inf   0.02208    0.0505
##  Isonet-LCG BIOX235 500 d/ha G2 Lb  0.0478 9.11e-03 Inf   0.03277    0.0692
##  Untreated                   G2 Lb  0.2751 2.99e-02 Inf   0.22049    0.3374
##  Isonet-LCG BIOX235 500 d/ha G3 Lb  0.0374 9.60e-03 Inf   0.02250    0.0615
##  Untreated                   G3 Lb  0.2624 3.25e-02 Inf   0.20376    0.3310
##  Isonet-LCG BIOX235 500 d/ha G3 zCg 0.0000 8.40e-07 Inf   0.00000    1.0000
##  Untreated                   G3 zCg 0.0090 4.20e-03 Inf   0.00359    0.0223
## 
## Confidence level used: 0.95 
## Intervals are back-transformed from the logit scale
```

```
test(pairs(e, simple = "Treatment"), by = NULL, adjust = "bonferroni")
```

```
##  contrast                                  Gen    odds.ratio       SE  df null
##  (Isonet-LCG BIOX235 500 d/ha) / Untreated G1 Lb       0.407 1.49e-01 Inf    1
##  (Isonet-LCG BIOX235 500 d/ha) / Untreated G2 Lb       0.132 3.30e-02 Inf    1
##  (Isonet-LCG BIOX235 500 d/ha) / Untreated G3 Lb       0.109 3.43e-02 Inf    1
##  (Isonet-LCG BIOX235 500 d/ha) / Untreated G3 zCg      0.000 9.23e-05 Inf    1
##  z.ratio p.value
##   -2.459  0.0558
##   -8.101  <.0001
##   -7.042  <.0001
##   -0.008  1.0000
## 
## P value adjustment: bonferroni method for 4 tests 
## Tests are performed on the log odds ratio scale
```

**Plot**

```
toplot <- as.data.frame(e)

ggplot(toplot, aes(x=Gen, y=prob*100, fill=Treatment))+
  geom_bar(stat="identity", position=position_dodge())+
  geom_errorbar(aes(ymin=(prob-SE)*100, ymax=(prob+SE)*100), position = position_dodge(.9), width=0.2)+
  labs(title = "Tuscany 2022 (Viognier)", x= 'Species', y='Infested bunches (%)')+
  theme(plot.title = element_text(hjust = 0.5))+
scale_fill_manual(values = c('#333333', '#BDBEBE'))+
  scale_x_discrete(labels=c('G1 Lb', 'G2 Lb', 'G3 Lb', 'G3 Cg'))+
  ylim(NA,32)
```

**Number of nest per bunches**

```
inputV <- na.omit(inputV)

inputV$n.nests.bunch <- (inputV$n.nests.bunch)*(inputV$N..sampled.bunches)

tab2 <- data.frame(Treatment=character(),
                 Sb=integer(), 
                 Gen=character(),
                 Bin=integer(),
                 stringsAsFactors=FALSE)

for (i in 1:length(inputV$Treatment)){
  n.bunches <- inputV$N..sampled.bunches[i]
  Treatment <- rep(inputV$Treatment[i], n.bunches)
  Sb <- rep(inputV$rep[i], n.bunches)
  Gen <- rep(inputV$Gen[i], n.bunches)
  Bin <- c(rep(1, inputV$n.nests.bunch[i]), rep(0, n.bunches - inputV$n.nests.bunch[i]))
  toadd <- data.frame(Treatment, Sb, Gen, Bin)
  tab2 <- rbind(tab2, toadd)
}

tab2$rand <- paste("plot", tab2$Sb, tab2$Treatment, tab2$Gen)

tab2$rand <- as.factor(tab2$rand)
tab2$Treatment <- as.factor(tab2$Treatment)
```

**Model**

```
modelN2 <- glmmTMB(Bin ~Treatment*Gen + (1|rand), data=tab2, family=binomial())
```

```
Anova(modelN2)
```

```
## Analysis of Deviance Table (Type II Wald chisquare tests)
## 
## Response: Bin
##                  Chisq Df Pr(>Chisq)    
## Treatment     120.7967  1  < 2.2e-16 ***
## Gen            96.7286  2  < 2.2e-16 ***
## Treatment:Gen   9.3387  2   0.009378 ** 
## ---
## Signif. codes:  0 '***' 0.001 '**' 0.01 '*' 0.05 '.' 0.1 ' ' 1
```

```
e <- emmeans(modelN2, ~Treatment*Gen, type='response')
e
```

```
##  Treatment                   Gen     prob      SE  df asymp.LCL asymp.UCL
##  Isonet-LCG BIOX235 500 d/ha G1 Lb 0.0147 0.00422 Inf   0.00836    0.0257
##  Untreated                   G1 Lb 0.0379 0.00786 Inf   0.02514    0.0566
##  Isonet-LCG BIOX235 500 d/ha G2 Lb 0.0480 0.00937 Inf   0.03263    0.0701
##  Untreated                   G2 Lb 0.3086 0.03308 Inf   0.24778    0.3769
##  Isonet-LCG BIOX235 500 d/ha G3 Lb 0.0371 0.00968 Inf   0.02219    0.0615
##  Untreated                   G3 Lb 0.2768 0.03452 Inf   0.21446    0.3492
## 
## Confidence level used: 0.95 
## Intervals are back-transformed from the logit scale
```

```
test(pairs(e, simple = "Treatment"), by = NULL, adjust = "bonferroni")
```

```
##  contrast                                  Gen   odds.ratio     SE  df null
##  (Isonet-LCG BIOX235 500 d/ha) / Untreated G1 Lb      0.379 0.1368 Inf    1
##  (Isonet-LCG BIOX235 500 d/ha) / Untreated G2 Lb      0.113 0.0290 Inf    1
##  (Isonet-LCG BIOX235 500 d/ha) / Untreated G3 Lb      0.101 0.0323 Inf    1
##  z.ratio p.value
##   -2.687  0.0216
##   -8.500  <.0001
##   -7.166  <.0001
## 
## P value adjustment: bonferroni method for 3 tests 
## Tests are performed on the log odds ratio scale
```

```
toplot <- as.data.frame(e)


ggplot(toplot, aes(x=Gen, y=prob*100, fill=Treatment))+
geom_bar(stat="identity", position=position_dodge())+
  geom_errorbar(aes(ymin=(prob-SE)*100, ymax=(prob+SE)*100), position = position_dodge(.9), width=0.2)+
  labs(title = "Tuscany 2022 (Viognier)", x= 'Species', y='Nest per bunches (%)')+
  theme(plot.title = element_text(hjust = 0.5))+
scale_fill_manual(values = c('#333333', '#BDBEBE'))+
  scale_x_discrete(labels=c('G1 Lb', 'G2 Lb', 'G3 Lb'))
```

## 2.2 Apulia

### 2.2.1 Aglianico 2022

**Load data**

```
inputA <- read.csv2(("analysis_aglianico.csv"))
inputA <- subset(inputA,Treatment!='Isonet L TT 250d/ha + MISTER V 3d/ha')
kable(head(inputA))
```

| Treatment | Subplot | N..sampled.bunches | N..infested.bunches | X..inf.bunches | Gen | Species | n.nests.bunch |
| --- | --- | --- | --- | --- | --- | --- | --- |
| Untreated | 1 | 50 | 16 | 32 | G3 zCg | Cg | NA |
| Untreated | 2 | 50 | 18 | 36 | G3 zCg | Cg | NA |
| Untreated | 3 | 50 | 13 | 26 | G3 zCg | Cg | NA |
| Untreated | 4 | 50 | 17 | 34 | G3 zCg | Cg | NA |
| Untreated | 5 | 50 | 2 | 4 | G3 zCg | Cg | NA |
| Untreated | 6 | 50 | 21 | 42 | G3 zCg | Cg | NA |

```
tab3 <- data.frame(Treatment=character(),
                 Sb=integer(), 
                 Gen=character(), 
                 Species=character(),
                 Binary=integer(),
                 stringsAsFactors=FALSE)

for (i in 1:length(inputA$Treatment)){
  n.bunches <- inputA$N..sampled.bunches[i]
  Treatment <- rep(inputA$Treatment[i], n.bunches)
  Sb <- rep(inputA$Subplot[i], n.bunches)
  Binary <- c(rep(1, inputA$N..infested.bunches[i]), rep(0, n.bunches - inputA$N..infested.bunches[i]))
  Gen <- rep(inputA$Gen[i], n.bunches)
  if(Gen[1]=="G3 zCg"){Species <- rep("Cg", n.bunches)} else {Species <- rep("Lb", n.bunches)}
  toadd <- data.frame(Treatment, Sb, Gen, Species, Binary)
  tab3 <- rbind(tab3, toadd)
}

tab3$onlygen <- tab3$Gen
tab3$onlygen[tab3$onlygen == "G3 Lb"] <-"G3"
tab3$onlygen[tab3$onlygen == "G3 zCg"] <-"G3"
tab3$onlygen[tab3$onlygen == "G1 Lb"] <-"G1"
tab3$onlygen[tab3$onlygen == "G2 Lb"] <-"G2"

tab3$rand <- paste("plot", tab3$Sb, tab3$Treatment, tab3$onlygen)

tab3$rand <- as.factor(tab3$rand)
tab3$Treatment <- as.factor(tab3$Treatment)
```

**Model**

```
model3 <- glmmTMB(Binary ~Treatment*Gen + (1|rand), data=tab3, family=binomial())
```

```
Anova(model3)
```

```
## Analysis of Deviance Table (Type II Wald chisquare tests)
## 
## Response: Binary
##                 Chisq Df Pr(>Chisq)    
## Treatment     107.690  2  < 2.2e-16 ***
## Gen           188.286  3  < 2.2e-16 ***
## Treatment:Gen  50.629  6  3.517e-09 ***
## ---
## Signif. codes:  0 '***' 0.001 '**' 0.01 '*' 0.05 '.' 0.1 ' ' 1
```

```
e <- emmeans(model3, ~Treatment*Gen, type='response')
e
```

```
##  Treatment                   Gen       prob      SE  df asymp.LCL asymp.UCL
##  Isonet-LCG BIOX235 400 d/ha G1 Lb  0.00190 0.00136 Inf  0.000467   0.00768
##  Isonet-LCG BIOX235 500 d/ha G1 Lb  0.00190 0.00136 Inf  0.000467   0.00768
##  Untreated                   G1 Lb  0.00759 0.00280 Inf  0.003679   0.01558
##  Isonet-LCG BIOX235 400 d/ha G2 Lb  0.05396 0.00887 Inf  0.038995   0.07422
##  Isonet-LCG BIOX235 500 d/ha G2 Lb  0.01944 0.00475 Inf  0.012016   0.03130
##  Untreated                   G2 Lb  0.19698 0.02076 Inf  0.159433   0.24084
##  Isonet-LCG BIOX235 400 d/ha G3 Lb  0.08505 0.01480 Inf  0.060179   0.11891
##  Isonet-LCG BIOX235 500 d/ha G3 Lb  0.03233 0.00843 Inf  0.019329   0.05361
##  Untreated                   G3 Lb  0.20185 0.02457 Inf  0.157946   0.25428
##  Isonet-LCG BIOX235 400 d/ha G3 zCg 0.11249 0.01750 Inf  0.082486   0.15161
##  Isonet-LCG BIOX235 500 d/ha G3 zCg 0.16488 0.02197 Inf  0.126189   0.21256
##  Untreated                   G3 zCg 0.23389 0.02659 Inf  0.185832   0.28996
## 
## Confidence level used: 0.95 
## Intervals are back-transformed from the logit scale
```

```
test(pairs(e, simple = "Treatment"), by = NULL, adjust = "bonferroni")
```

```
##  contrast                                                      Gen   
##  (Isonet-LCG BIOX235 400 d/ha) / (Isonet-LCG BIOX235 500 d/ha) G1 Lb 
##  (Isonet-LCG BIOX235 400 d/ha) / Untreated                     G1 Lb 
##  (Isonet-LCG BIOX235 500 d/ha) / Untreated                     G1 Lb 
##  (Isonet-LCG BIOX235 400 d/ha) / (Isonet-LCG BIOX235 500 d/ha) G2 Lb 
##  (Isonet-LCG BIOX235 400 d/ha) / Untreated                     G2 Lb 
##  (Isonet-LCG BIOX235 500 d/ha) / Untreated                     G2 Lb 
##  (Isonet-LCG BIOX235 400 d/ha) / (Isonet-LCG BIOX235 500 d/ha) G3 Lb 
##  (Isonet-LCG BIOX235 400 d/ha) / Untreated                     G3 Lb 
##  (Isonet-LCG BIOX235 500 d/ha) / Untreated                     G3 Lb 
##  (Isonet-LCG BIOX235 400 d/ha) / (Isonet-LCG BIOX235 500 d/ha) G3 zCg
##  (Isonet-LCG BIOX235 400 d/ha) / Untreated                     G3 zCg
##  (Isonet-LCG BIOX235 500 d/ha) / Untreated                     G3 zCg
##  odds.ratio     SE  df null z.ratio p.value
##      1.0000 1.0122 Inf    1   0.000  1.0000
##      0.2489 0.2006 Inf    1  -1.725  1.0000
##      0.2489 0.2006 Inf    1  -1.725  1.0000
##      2.8771 0.8695 Inf    1   3.497  0.0057
##      0.2325 0.0505 Inf    1  -6.715  <.0001
##      0.0808 0.0227 Inf    1  -8.964  <.0001
##      2.7821 0.9158 Inf    1   3.109  0.0226
##      0.3676 0.0895 Inf    1  -4.110  0.0005
##      0.1321 0.0408 Inf    1  -6.547  <.0001
##      0.6420 0.1518 Inf    1  -1.874  0.7312
##      0.4152 0.0952 Inf    1  -3.832  0.0015
##      0.6467 0.1407 Inf    1  -2.003  0.5423
## 
## P value adjustment: bonferroni method for 12 tests 
## Tests are performed on the log odds ratio scale
```

**Plot**

```
toplot <- as.data.frame(e)

ggplot(toplot, aes(x=Gen, y=prob*100, fill=Treatment))+
  geom_bar(stat="identity", position=position_dodge())+
  geom_errorbar(aes(ymin=(prob-SE)*100, ymax=(prob+SE)*100), position = position_dodge(.9), width=0.2)+
  labs(title = "Apulia 2022 (Aglianico)", x= 'Species', y='Infested bunches (%)')+
  theme(plot.title = element_text(hjust = 0.5))+
scale_fill_manual(values = c('#6E6E6E','#333333', '#BDBEBE'))+
  scale_x_discrete(labels=c('G1 Lb', 'G2 Lb', 'G3 Lb', 'G3 Cg'))
```

**Number of nest per bunches**

```
inputA <- na.omit(inputA)
inputA$n.nests.bunch <- (inputA$n.nests.bunch)*(inputA$N..sampled.bunches)

tab3 <- data.frame(Treatment=character(),
                 Sb=integer(), 
                 Gen=character(),
                 Bin=integer(),
                 stringsAsFactors=FALSE)

for (i in 1:length(inputA$Treatment)){
  n.bunches <- inputA$N..sampled.bunches[i]
  Treatment <- rep(inputA$Treatment[i], n.bunches)
  Sb <- rep(inputA$Subplot[i], n.bunches)
  Gen <- rep(inputA$Gen[i], n.bunches)
  Bin <- c(rep(1, inputA$n.nests.bunch[i]), rep(0, n.bunches - inputA$n.nests.bunch[i]))
  toadd <- data.frame(Treatment, Sb, Gen, Bin)
  tab3 <- rbind(tab3, toadd)
}

tab3$rand <- paste("plot", tab3$Sb, tab3$Treatment, tab3$Gen)

tab3$rand <- as.factor(tab3$rand)
tab3$Treatment <- as.factor(tab3$Treatment)
```

**Model**

```
modelN3 <- glmmTMB(Bin ~Treatment*Gen + (1|rand), data=tab3, family=binomial())
```

```
Anova(modelN3)
```

```
## Analysis of Deviance Table (Type II Wald chisquare tests)
## 
## Response: Bin
##                  Chisq Df Pr(>Chisq)    
## Treatment     163.3138  2     <2e-16 ***
## Gen           131.6809  2     <2e-16 ***
## Treatment:Gen   1.6976  4     0.7912    
## ---
## Signif. codes:  0 '***' 0.001 '**' 0.01 '*' 0.05 '.' 0.1 ' ' 1
```

```
e <- emmeans(modelN3, ~Treatment*Gen, type='response')
e
```

```
##  Treatment                   Gen       prob       SE  df asymp.LCL asymp.UCL
##  Isonet-LCG BIOX235 400 d/ha G1 Lb 0.001904 0.001360 Inf  0.000469    0.0077
##  Isonet-LCG BIOX235 500 d/ha G1 Lb 0.000952 0.000957 Inf  0.000133    0.0068
##  Untreated                   G1 Lb 0.007609 0.002799 Inf  0.003694    0.0156
##  Isonet-LCG BIOX235 400 d/ha G2 Lb 0.054048 0.008803 Inf  0.039174    0.0741
##  Isonet-LCG BIOX235 500 d/ha G2 Lb 0.019537 0.004772 Inf  0.012081    0.0314
##  Untreated                   G2 Lb 0.197156 0.020425 Inf  0.160155    0.2403
##  Isonet-LCG BIOX235 400 d/ha G3 Lb 0.094947 0.015704 Inf  0.068316    0.1305
##  Isonet-LCG BIOX235 500 d/ha G3 Lb 0.034423 0.008727 Inf  0.020865    0.0563
##  Untreated                   G3 Lb 0.238097 0.026501 Inf  0.190083    0.2938
## 
## Confidence level used: 0.95 
## Intervals are back-transformed from the logit scale
```

```
test(pairs(e, simple = "Treatment"), by = NULL, adjust = "bonferroni")
```

```
##  contrast                                                      Gen   odds.ratio
##  (Isonet-LCG BIOX235 400 d/ha) / (Isonet-LCG BIOX235 500 d/ha) G1 Lb     2.0024
##  (Isonet-LCG BIOX235 400 d/ha) / Untreated                     G1 Lb     0.2488
##  (Isonet-LCG BIOX235 500 d/ha) / Untreated                     G1 Lb     0.1243
##  (Isonet-LCG BIOX235 400 d/ha) / (Isonet-LCG BIOX235 500 d/ha) G2 Lb     2.8673
##  (Isonet-LCG BIOX235 400 d/ha) / Untreated                     G2 Lb     0.2327
##  (Isonet-LCG BIOX235 500 d/ha) / Untreated                     G2 Lb     0.0811
##  (Isonet-LCG BIOX235 400 d/ha) / (Isonet-LCG BIOX235 500 d/ha) G3 Lb     2.9427
##  (Isonet-LCG BIOX235 400 d/ha) / Untreated                     G3 Lb     0.3357
##  (Isonet-LCG BIOX235 500 d/ha) / Untreated                     G3 Lb     0.1141
##      SE  df null z.ratio p.value
##  2.4714 Inf    1   0.563  1.0000
##  0.2004 Inf    1  -1.727  0.7568
##  0.1332 Inf    1  -1.946  0.4650
##  0.8613 Inf    1   3.507  0.0041
##  0.0499 Inf    1  -6.801  <.0001
##  0.0226 Inf    1  -9.006  <.0001
##  0.9380 Inf    1   3.386  0.0064
##  0.0784 Inf    1  -4.676  <.0001
##  0.0342 Inf    1  -7.241  <.0001
## 
## P value adjustment: bonferroni method for 9 tests 
## Tests are performed on the log odds ratio scale
```

```
toplot <- as.data.frame(e)


ggplot(toplot, aes(x=Gen, y=prob*100, fill=Treatment))+
geom_bar(stat="identity", position=position_dodge())+
  geom_errorbar(aes(ymin=(prob-SE)*100, ymax=(prob+SE)*100), position = position_dodge(.9), width=0.2)+
  labs(title = "Tuscany 2022 (Viognier)", x= 'Species', y='Nest per bunches (%)')+
  theme(plot.title = element_text(hjust = 0.5))+
scale_fill_manual(values = c('#6E6E6E','#333333', '#BDBEBE'))+
  scale_x_discrete(labels=c('G1 Lb', 'G2 Lb', 'G3 Lb'))
```

**Flights**

```
inputC <- read.csv2(("monitoraggio_crypto_22.csv"))

hist(inputC$pop, breaks = 20)
```

```
mp <- glmmTMB(pop ~Treatment +(1|Date), data =inputC, ziformula = ~1, family=poisson())
simres <- simulateResiduals(mp)
plot(simres)
```

```
Anova(mp)
```

```
## Analysis of Deviance Table (Type II Wald chisquare tests)
## 
## Response: pop
##            Chisq Df Pr(>Chisq)   
## Treatment 12.856  2   0.001616 **
## ---
## Signif. codes:  0 '***' 0.001 '**' 0.01 '*' 0.05 '.' 0.1 ' ' 1
```

```
e <- emmeans(mp, ~Treatment, type='response')
e
```

```
##  Treatment    rate     SE  df asymp.LCL asymp.UCL
##  Ctrl      0.02049 0.0516 Inf  1.47e-04      2.86
##  LCGX 400  0.00468 0.0119 Inf  3.23e-05      0.68
##  LCGX 500  0.00904 0.0227 Inf  6.55e-05      1.25
## 
## Confidence level used: 0.95 
## Intervals are back-transformed from the log scale
```

```
pairs(e)
```

```
##  contrast            ratio    SE  df null z.ratio p.value
##  Ctrl / LCGX 400     4.376 1.979 Inf    1   3.265  0.0031
##  Ctrl / LCGX 500     2.267 0.792 Inf    1   2.344  0.0500
##  LCGX 400 / LCGX 500 0.518 0.252 Inf    1  -1.351  0.3672
## 
## P value adjustment: tukey method for comparing a family of 3 estimates 
## Tests are performed on the log scale
```

```
inputC$Date <- as.Date(inputC$Date, format= "%d/%m/%Y")

ggplot(inputC, aes(x=Date, y=pop, group=Treatment, color=Treatment))+
  geom_point()+
  geom_line(aes(linetype=Treatment))+
    scale_color_manual(values = c('#6E6E6E','#333333', '#BDBEBE'))+
  theme(axis.text.x = element_text(angle = 90, vjust = 0.5, hjust=1))+
  scale_x_date(date_labels = "%d-%m-%Y", breaks = unique(inputC$Date))
```

### 2.2.2 Aglianico 2023

**Load data**

```
inputG <- read.csv2(("analysis_bdl_23.csv"))
kable(head(inputG))
```

| tesi | rep | N..sampled.bunches | N..infested.bunches | P\_inf | Gen | n.nests.bunch |
| --- | --- | --- | --- | --- | --- | --- |
| Untreated | 1 | 100 | 4 | 4 | G1 Lb | 0.04 |
| Untreated | 2 | 100 | 1 | 1 | G1 Lb | 0.03 |
| Untreated | 3 | 100 | 6 | 6 | G1 Lb | 0.07 |
| Untreated | 4 | 100 | 14 | 14 | G1 Lb | 0.17 |
| Untreated | 5 | 100 | 8 | 8 | G1 Lb | 0.10 |
| Untreated | 6 | 100 | 11 | 11 | G1 Lb | 0.14 |

```
tab4 <- data.frame(Treatment=character(),
                 Sb=integer(), 
                 Gen=character(), 
                 Species=character(),
                 Binary=integer(),
                 stringsAsFactors=FALSE)

for (i in 1:length(inputG$tesi)){
  n.bunches <- inputG$N..sampled.bunches[i]
  Treatment <- rep(inputG$tesi[i], n.bunches)
  Sb <- rep(inputG$rep[i], n.bunches)
  Binary <- c(rep(1, inputG$N..infested.bunches[i]), rep(0, n.bunches - inputG$N..infested.bunches[i]))
  Gen <- rep(inputG$Gen[i], n.bunches)
  toadd <- data.frame(Treatment, Sb, Gen, Species, Binary)
  tab4 <- rbind(tab4, toadd)
}


tab4$rand <- paste("plot", tab4$Sb, tab4$Treatment, tab4$Gen)

tab4$rand <- as.factor(tab4$rand)
tab4$Treatment <- as.factor(tab4$Treatment)
```

**Model**

```
model4 <- glmmTMB(Binary ~Treatment*Gen + (1|rand), data=tab4, family=binomial())
```

```
Anova(model4)
```

```
## Analysis of Deviance Table (Type II Wald chisquare tests)
## 
## Response: Binary
##                 Chisq Df Pr(>Chisq)    
## Treatment     24.7985  1  6.365e-07 ***
## Gen            1.3347  1     0.2480    
## Treatment:Gen  1.9722  1     0.1602    
## ---
## Signif. codes:  0 '***' 0.001 '**' 0.01 '*' 0.05 '.' 0.1 ' ' 1
```

```
e <- emmeans(model4, ~Treatment*Gen, type='response')
e
```

```
##  Treatment                   Gen     prob      SE  df asymp.LCL asymp.UCL
##  Isonet-LCG BIOX235 500 d/ha G1 Lb 0.0120 0.00412 Inf   0.00613    0.0235
##  Untreated                   G1 Lb 0.0739 0.01626 Inf   0.04771    0.1128
##  Isonet-LCG BIOX235 500 d/ha G2 Lb 0.0147 0.00481 Inf   0.00771    0.0278
##  Untreated                   G2 Lb 0.0411 0.01054 Inf   0.02470    0.0675
## 
## Confidence level used: 0.95 
## Intervals are back-transformed from the logit scale
```

```
test(pairs(e, simple = "Treatment"), by = NULL, adjust = "bonferroni")
```

```
##  contrast                                  Gen   odds.ratio     SE  df null
##  (Isonet-LCG BIOX235 500 d/ha) / Untreated G1 Lb      0.153 0.0637 Inf    1
##  (Isonet-LCG BIOX235 500 d/ha) / Untreated G2 Lb      0.348 0.1440 Inf    1
##  z.ratio p.value
##   -4.505  <.0001
##   -2.550  0.0215
## 
## P value adjustment: bonferroni method for 2 tests 
## Tests are performed on the log odds ratio scale
```

**Plot**

```
toplot <- as.data.frame(e)

ggplot(toplot, aes(x=Gen, y=prob*100, fill=Treatment))+
  geom_bar(stat="identity", position=position_dodge())+
  geom_errorbar(aes(ymin=(prob-SE)*100, ymax=(prob+SE)*100), position = position_dodge(.9), width=0.2)+
  labs(title = "Apulia 2023 (Aglianico)", x= 'Species', y='Infested bunches (%)')+
  theme(plot.title = element_text(hjust = 0.5))+
scale_fill_manual(values = c('#333333', '#BDBEBE'))+
  ylim(0,10)
```

**Number of nest per bunches**

```
inputG$n.nests.bunch <- (inputG$n.nests.bunch)*(inputG$N..sampled.bunches)

tab4 <- data.frame(Treatment=character(),
                 Sb=integer(), 
                 Gen=character(),
                 Bin=integer(),
                 stringsAsFactors=FALSE)

for (i in 1:length(inputG$tesi)){
  n.bunches <- inputG$N..sampled.bunches[i]
  Treatment <- rep(inputG$tesi[i], n.bunches)
  Sb <- rep(inputG$rep[i], n.bunches)
  Gen <- rep(inputG$Gen[i], n.bunches)
  Bin <- c(rep(1, inputG$n.nests.bunch[i]), rep(0, n.bunches - inputG$n.nests.bunch[i]))
  toadd <- data.frame(Treatment, Sb, Gen, Bin)
  tab4 <- rbind(tab4, toadd)
}

tab4$rand <- paste("plot", tab4$Sb, tab4$Treatment, tab4$Gen)

tab4$rand <- as.factor(tab4$rand)
tab4$Treatment <- as.factor(tab4$Treatment)
```

**Model**

```
modelN4 <- glmmTMB(Bin ~Treatment*Gen + (1|rand), data=tab4, family=binomial())
```

```
Anova(modelN4)
```

```
## Analysis of Deviance Table (Type II Wald chisquare tests)
## 
## Response: Bin
##                 Chisq Df Pr(>Chisq)    
## Treatment     38.6569  1  5.053e-10 ***
## Gen            1.6102  1     0.2045    
## Treatment:Gen  0.6530  1     0.4191    
## ---
## Signif. codes:  0 '***' 0.001 '**' 0.01 '*' 0.05 '.' 0.1 ' ' 1
```

```
e <- emmeans(modelN4, ~Treatment*Gen, type='response')
e
```

```
##  Treatment                   Gen     prob      SE  df asymp.LCL asymp.UCL
##  Isonet-LCG BIOX235 500 d/ha G1 Lb 0.0164 0.00462 Inf   0.00944    0.0284
##  Untreated                   G1 Lb 0.0931 0.01540 Inf   0.06700    0.1280
##  Isonet-LCG BIOX235 500 d/ha G2 Lb 0.0164 0.00601 Inf   0.00795    0.0335
##  Untreated                   G2 Lb 0.0616 0.01393 Inf   0.03937    0.0953
## 
## Confidence level used: 0.95 
## Intervals are back-transformed from the logit scale
```

```
test(pairs(e, simple = "Treatment"), by = NULL, adjust = "bonferroni")
```

```
##  contrast                                  Gen   odds.ratio     SE  df null
##  (Isonet-LCG BIOX235 500 d/ha) / Untreated G1 Lb      0.163 0.0545 Inf    1
##  (Isonet-LCG BIOX235 500 d/ha) / Untreated G2 Lb      0.254 0.1105 Inf    1
##  z.ratio p.value
##   -5.421  <.0001
##   -3.147  0.0033
## 
## P value adjustment: bonferroni method for 2 tests 
## Tests are performed on the log odds ratio scale
```

```
toplot <- as.data.frame(e)


ggplot(toplot, aes(x=Gen, y=prob*100, fill=Treatment))+
geom_bar(stat="identity", position=position_dodge())+
  geom_errorbar(aes(ymin=(prob-SE)*100, ymax=(prob+SE)*100), position = position_dodge(.9), width=0.2)+
  labs(title = "Apulia 2023 (Aglianico)", x= 'Species', y='Nest per bunches (%)')+
  theme(plot.title = element_text(hjust = 0.5))+
scale_fill_manual(values = c('#333333', '#BDBEBE'))+
  scale_x_discrete(labels=c('G1 Lb', 'G2 Lb'))
```

```
inputB <- read.csv2(("analysis_aglianico_23.csv"))
kable(head(inputB))
```

| Treatment | Subplot | N..sampled.bunches | N..infested.bunches | P\_inf | Gen | Species | Bt | n.nests.bunch |
| --- | --- | --- | --- | --- | --- | --- | --- | --- |
| Untreated | 1 | 50 | 2 | 4 | G3 zCg | Cg | 0 | NA |
| Untreated | 2 | 50 | 2 | 4 | G3 zCg | Cg | 0 | NA |
| Untreated | 3 | 50 | 1 | 2 | G3 zCg | Cg | 0 | NA |
| Untreated | 4 | 50 | 2 | 4 | G3 zCg | Cg | 0 | NA |
| Untreated | 5 | 50 | 3 | 6 | G3 zCg | Cg | 0 | NA |
| Untreated | 6 | 50 | 6 | 12 | G3 zCg | Cg | 0 | NA |

```
tab5 <- data.frame(Treatment=character(),
                 Sb=integer(), 
                 Gen=character(), 
                 Species=character(),
                 Binary=integer(),
                 stringsAsFactors=FALSE)

for (i in 1:length(inputB$Treatment)){
  n.bunches <- inputB$N..sampled.bunches[i]
  Treatment <- rep(inputB$Treatment[i], n.bunches)
  Sb <- rep(inputB$Subplot[i], n.bunches)
  Binary <- c(rep(1, inputB$N..infested.bunches[i]), rep(0, n.bunches - inputB$N..infested.bunches[i]))
  Gen <- rep(inputB$Gen[i], n.bunches)
  if(Gen[1]=="G3 zCg"){Species <- rep("Cg", n.bunches)} else {Species <- rep("Lb", n.bunches)}
  toadd <- data.frame(Treatment, Sb, Gen, Species, Binary)
  tab5 <- rbind(tab5, toadd)
}

tab5$rand <- paste("plot", tab5$Sb, tab5$Treatment, tab5$onlygen)

tab5$rand <- as.factor(tab5$rand)
tab5$Treatment <- as.factor(tab5$Treatment)
```

**Model**

```
model5 <- glmmTMB(Binary ~Treatment*Gen + (1|rand), data=tab5, family=binomial())
```

```
Anova(model5)
```

```
## Analysis of Deviance Table (Type II Wald chisquare tests)
## 
## Response: Binary
##                  Chisq Df Pr(>Chisq)    
## Treatment       3.3281  2    0.18937    
## Gen           164.3415  1    < 2e-16 ***
## Treatment:Gen   8.7340  2    0.01269 *  
## ---
## Signif. codes:  0 '***' 0.001 '**' 0.01 '*' 0.05 '.' 0.1 ' ' 1
```

```
e <- emmeans(model5, ~Treatment*Gen, type='response')
e
```

```
##  Treatment                        Gen      prob      SE  df asymp.LCL asymp.UCL
##  Bt                               G3 Lb  0.2485 0.02580 Inf   0.20138    0.3024
##  Isonet-LCG BIOX235 500 d/ha + Bt G3 Lb  0.1840 0.02214 Inf   0.14453    0.2314
##  Untreated                        G3 Lb  0.2220 0.02442 Inf   0.17780    0.2735
##  Bt                               G3 zCg 0.0174 0.00596 Inf   0.00884    0.0339
##  Isonet-LCG BIOX235 500 d/ha + Bt G3 zCg 0.0289 0.00782 Inf   0.01694    0.0489
##  Untreated                        G3 zCg 0.0501 0.01059 Inf   0.03300    0.0755
## 
## Confidence level used: 0.95 
## Intervals are back-transformed from the logit scale
```

```
test(pairs(e, simple = "Treatment"), by = NULL, adjust = "bonferroni")
```

```
##  contrast                                       Gen    odds.ratio    SE  df
##  Bt / (Isonet-LCG BIOX235 500 d/ha + Bt)        G3 Lb       1.466 0.295 Inf
##  Bt / Untreated                                 G3 Lb       1.159 0.229 Inf
##  (Isonet-LCG BIOX235 500 d/ha + Bt) / Untreated G3 Lb       0.791 0.161 Inf
##  Bt / (Isonet-LCG BIOX235 500 d/ha + Bt)        G3 zCg      0.594 0.265 Inf
##  Bt / Untreated                                 G3 zCg      0.335 0.138 Inf
##  (Isonet-LCG BIOX235 500 d/ha + Bt) / Untreated G3 zCg      0.564 0.201 Inf
##  null z.ratio p.value
##     1   1.897  0.3469
##     1   0.747  1.0000
##     1  -1.154  1.0000
##     1  -1.167  1.0000
##     1  -2.646  0.0489
##     1  -1.611  0.6427
## 
## P value adjustment: bonferroni method for 6 tests 
## Tests are performed on the log odds ratio scale
```

**Plot**

```
toplot <- as.data.frame(e)

ggplot(toplot, aes(x=Gen, y=prob*100, fill=Treatment))+
  geom_bar_pattern(stat="identity", position = position_dodge(),
                   pattern_color = "#8E8D8D",
                   pattern_fill = "#8E8D8D",
                   aes(pattern = Treatment))+
  geom_errorbar(aes(ymin=(prob-SE)*100, ymax=(prob+SE)*100), position = position_dodge(.9), width=0.2)+
  labs(title = "Apulia 2023 (Aglianico)", x= 'Species', y='Infested bunches (%)')+
  theme(plot.title = element_text(hjust = 0.5))+
scale_fill_manual(values = c('#8E8D8D','#333333', '#BDBEBE'))+
  scale_pattern_manual(values=c('none', 'stripe', 'none'))+
  scale_x_discrete(labels=c('G3 Lb', 'G3 Cg'))+
  ylim(0,30)
```

**Number of nest per bunches**

```
inputB <- na.omit(inputB)

inputB$n.nests.bunch <- (inputB$n.nests.bunch)*(inputB$N..sampled.bunches)

tab5 <- data.frame(Treatment=character(),
                 Sb=integer(), 
                 Gen=character(),
                 Bin=integer(),
                 stringsAsFactors=FALSE)

for (i in 1:length(inputB$Treatment)){
  n.bunches <- inputB$N..sampled.bunches[i]
  Treatment <- rep(inputB$Treatment[i], n.bunches)
  Sb <- rep(inputB$Subplot[i], n.bunches)
  Gen <- rep(inputB$Gen[i], n.bunches)
  Bin <- c(rep(1, inputB$n.nests.bunch[i]), rep(0, n.bunches - inputB$n.nests.bunch[i]))
  toadd <- data.frame(Treatment, Sb, Gen, Bin)
  tab5 <- rbind(tab5, toadd)
}

tab5$rand <- paste("plot", tab5$Sb, tab5$Treatment, tab5$n.bunches)

tab5$rand <- as.factor(tab5$rand)
tab5$Treatment <- as.factor(tab5$Treatment)
```

**Model**

```
modelN5 <- glmmTMB(Bin ~Treatment + (1|rand), data=tab5, family=binomial())
```

```
Anova(modelN5)
```

```
## Analysis of Deviance Table (Type II Wald chisquare tests)
## 
## Response: Bin
##            Chisq Df Pr(>Chisq)
## Treatment 4.3499  2     0.1136
```

```
e <- emmeans(modelN5, ~Treatment, type='response')
e
```

```
##  Treatment                         prob     SE  df asymp.LCL asymp.UCL
##  Bt                               0.290 0.0314 Inf     0.233     0.355
##  Isonet-LCG BIOX235 500 d/ha + Bt 0.208 0.0264 Inf     0.161     0.264
##  Untreated                        0.269 0.0302 Inf     0.214     0.332
## 
## Confidence level used: 0.95 
## Intervals are back-transformed from the logit scale
```

```
test(pairs(e, simple = "Treatment"), by = NULL, adjust = "bonferroni")
```

```
##  contrast                                       odds.ratio    SE  df null
##  Bt / (Isonet-LCG BIOX235 500 d/ha + Bt)             1.559 0.344 Inf    1
##  Bt / Untreated                                      1.112 0.241 Inf    1
##  (Isonet-LCG BIOX235 500 d/ha + Bt) / Untreated      0.713 0.158 Inf    1
##  z.ratio p.value
##    2.010  0.1332
##    0.490  1.0000
##   -1.524  0.3828
## 
## P value adjustment: bonferroni method for 3 tests 
## Tests are performed on the log odds ratio scale
```

```
toplot <- as.data.frame(e)

ggplot(toplot, aes(x=Treatment, y=prob*100, fill=Treatment))+
  geom_bar_pattern(stat="identity", position = position_dodge(),
                   pattern_color = "#8E8D8D",
                   pattern_fill = "#8E8D8D",
                   aes(pattern = Treatment))+
  geom_errorbar(aes(ymin=(prob-SE)*100, ymax=(prob+SE)*100), position = position_dodge(.9), width=0.2)+
  labs(title = "Apulia 2023 (Aglianico)", x= 'Species', y='Nests per bunches(%)')+
  theme(plot.title = element_text(hjust = 0.5))+
scale_fill_manual(values = c('#8E8D8D','#333333', '#BDBEBE'))+
  scale_pattern_manual(values=c('none', 'stripe', 'none'))
```

**Flights**

```
inputF <- read.csv2(("monitoraggio_crypto_23.csv"))

hist(inputF$pop, breaks = 20)
```

```
mpp <- glmmTMB(pop ~Treatment , data =inputF, ziformula = ~1, family=poisson())
simres <- simulateResiduals(mpp)
plot(simres)
```

```
Anova(mpp)
```

```
## Analysis of Deviance Table (Type II Wald chisquare tests)
## 
## Response: pop
##           Chisq Df Pr(>Chisq)
## Treatment     0  1     0.9993
```

```
e <- emmeans(mpp, ~Treatment, type='response')
e
```

```
##  Treatment rate       SE  df asymp.LCL asymp.UCL
##  Ctrl      5.48 1.67e+00 Inf      3.01        10
##  LCGX 500  0.00 1.92e-05 Inf      0.00       Inf
## 
## Confidence level used: 0.95 
## Intervals are back-transformed from the log scale
```

```
pairs(e)
```

```
##  contrast           ratio       SE  df null z.ratio p.value
##  Ctrl / LCGX 500 7.42e+09 1.93e+14 Inf    1   0.001  0.9993
## 
## Tests are performed on the log scale
```

```
inputF$Date <- as.Date(inputF$Date, format= "%d/%m/%Y")

ggplot(inputF, aes(x=Date, y=pop, group=Treatment, color=Treatment))+
  geom_point()+
  geom_line(aes(linetype=Treatment))+
    scale_color_manual(values = c('#6E6E6E','#333333', '#BDBEBE'))+
  theme(axis.text.x = element_text(angle = 90, vjust = 0.5, hjust=1))+
  scale_x_date(date_labels = "%d-%m-%Y", breaks = unique(inputF$Date))
```

# 3 Session info

```
print(sessioninfo::session_info())
```

```
## ─ Session info ───────────────────────────────────────────────────────────────
##  setting  value
##  version  R version 4.3.2 (2023-10-31 ucrt)
##  os       Windows 10 x64 (build 19042)
##  system   x86_64, mingw32
##  ui       RTerm
##  language (EN)
##  collate  Italian_Italy.utf8
##  ctype    Italian_Italy.utf8
##  tz       Europe/Rome
##  date     2024-02-09
##  pandoc   3.1.1 @ C:/Program Files/RStudio/resources/app/bin/quarto/bin/tools/ (via rmarkdown)
## 
## ─ Packages ───────────────────────────────────────────────────────────────────
##  ! package      * version    date (UTC) lib source
##    abind          1.4-5      2016-07-21 [1] CRAN (R 4.3.1)
##    bookdown       0.37       2023-12-01 [1] CRAN (R 4.3.2)
##    boot           1.3-28.1   2022-11-22 [2] CRAN (R 4.3.2)
##    bslib          0.6.1      2023-11-28 [1] CRAN (R 4.3.2)
##    cachem         1.0.8      2023-05-01 [1] CRAN (R 4.3.2)
##    car          * 3.1-2      2023-03-30 [1] CRAN (R 4.3.2)
##    carData      * 3.0-5      2022-01-06 [1] CRAN (R 4.3.2)
##    class          7.3-22     2023-05-03 [2] CRAN (R 4.3.2)
##    classInt       0.4-10     2023-09-05 [1] CRAN (R 4.3.2)
##    cli            3.6.2      2023-12-11 [1] CRAN (R 4.3.2)
##    codetools      0.2-19     2023-02-01 [2] CRAN (R 4.3.2)
##    colorspace     2.1-0      2023-01-23 [1] CRAN (R 4.3.2)
##    DBI            1.2.1      2024-01-12 [1] CRAN (R 4.3.2)
##    DHARMa       * 0.4.6      2022-09-08 [1] CRAN (R 4.3.2)
##    digest         0.6.34     2024-01-11 [1] CRAN (R 4.3.2)
##    dplyr          1.1.4      2023-11-17 [1] CRAN (R 4.3.2)
##    e1071          1.7-14     2023-12-06 [1] CRAN (R 4.3.2)
##    emmeans      * 1.10.0     2024-01-23 [1] CRAN (R 4.3.2)
##    estimability   1.4.1      2022-08-05 [1] CRAN (R 4.3.1)
##    evaluate       0.23       2023-11-01 [1] CRAN (R 4.3.2)
##    fansi          1.0.6      2023-12-08 [1] CRAN (R 4.3.2)
##    farver         2.1.1      2022-07-06 [1] CRAN (R 4.3.2)
##    fastmap        1.1.1      2023-02-24 [1] CRAN (R 4.3.2)
##    gap            1.5-3      2023-08-26 [1] CRAN (R 4.3.2)
##    gap.datasets   0.0.6      2023-08-25 [1] CRAN (R 4.3.1)
##    generics       0.1.3      2022-07-05 [1] CRAN (R 4.3.2)
##    ggpattern    * 1.0.1      2022-11-09 [1] CRAN (R 4.3.2)
##    ggplot2      * 3.4.4      2023-10-12 [1] CRAN (R 4.3.2)
##    glmmTMB      * 1.1.8      2023-10-07 [1] CRAN (R 4.3.2)
##    glue           1.7.0      2024-01-09 [1] CRAN (R 4.3.2)
##    gridpattern    1.1.1      2023-10-25 [1] CRAN (R 4.3.2)
##    gtable         0.3.4      2023-08-21 [1] CRAN (R 4.3.2)
##    highr          0.10       2022-12-22 [1] CRAN (R 4.3.2)
##    htmltools      0.5.7      2023-11-03 [1] CRAN (R 4.3.2)
##    jquerylib      0.1.4      2021-04-26 [1] CRAN (R 4.3.2)
##    jsonlite       1.8.8      2023-12-04 [1] CRAN (R 4.3.2)
##    KernSmooth     2.23-22    2023-07-10 [2] CRAN (R 4.3.2)
##    knitr        * 1.45       2023-10-30 [1] CRAN (R 4.3.2)
##    labeling       0.4.3      2023-08-29 [1] CRAN (R 4.3.1)
##    lattice        0.21-9     2023-10-01 [2] CRAN (R 4.3.2)
##    lifecycle      1.0.4      2023-11-07 [1] CRAN (R 4.3.2)
##    lme4           1.1-35.1   2023-11-05 [1] CRAN (R 4.3.2)
##    magrittr       2.0.3      2022-03-30 [1] CRAN (R 4.3.2)
##    MASS           7.3-60     2023-05-04 [2] CRAN (R 4.3.2)
##    Matrix         1.6-5      2024-01-11 [1] CRAN (R 4.3.2)
##    memoise        2.0.1      2021-11-26 [1] CRAN (R 4.3.2)
##    mgcv           1.9-0      2023-07-11 [2] CRAN (R 4.3.2)
##    minqa          1.2.6      2023-09-11 [1] CRAN (R 4.3.2)
##    munsell        0.5.0      2018-06-12 [1] CRAN (R 4.3.2)
##    mvtnorm        1.2-4      2023-11-27 [1] CRAN (R 4.3.2)
##    nlme           3.1-163    2023-08-09 [2] CRAN (R 4.3.2)
##    nloptr         2.0.3      2022-05-26 [1] CRAN (R 4.3.2)
##    numDeriv       2016.8-1.1 2019-06-06 [1] CRAN (R 4.3.1)
##    pillar         1.9.0      2023-03-22 [1] CRAN (R 4.3.2)
##    pkgconfig      2.0.3      2019-09-22 [1] CRAN (R 4.3.2)
##    plyr           1.8.9      2023-10-02 [1] CRAN (R 4.3.2)
##    proxy          0.4-27     2022-06-09 [1] CRAN (R 4.3.2)
##    R6             2.5.1      2021-08-19 [1] CRAN (R 4.3.2)
##    rbibutils      2.2.16     2023-10-25 [1] CRAN (R 4.3.2)
##    Rcpp           1.0.12     2024-01-09 [1] CRAN (R 4.3.2)
##    Rdpack         2.6        2023-11-08 [1] CRAN (R 4.3.2)
##    reshape2     * 1.4.4      2020-04-09 [1] CRAN (R 4.3.2)
##    rlang          1.1.3      2024-01-10 [1] CRAN (R 4.3.2)
##    rmarkdown      2.25       2023-09-18 [1] CRAN (R 4.3.2)
##    rmdformats     1.0.4      2022-05-17 [1] CRAN (R 4.3.2)
##    sass           0.4.8      2023-12-06 [1] CRAN (R 4.3.2)
##    scales         1.3.0      2023-11-28 [1] CRAN (R 4.3.2)
##    sessioninfo    1.2.2      2021-12-06 [1] CRAN (R 4.3.2)
##    sf             1.0-15     2023-12-18 [1] CRAN (R 4.3.2)
##    stringi        1.8.3      2023-12-11 [1] CRAN (R 4.3.2)
##    stringr        1.5.1      2023-11-14 [1] CRAN (R 4.3.2)
##    tibble         3.2.1      2023-03-20 [1] CRAN (R 4.3.2)
##    tidyselect     1.2.0      2022-10-10 [1] CRAN (R 4.3.2)
##  D TMB            1.9.10     2023-12-12 [1] CRAN (R 4.3.2)
##    units          0.8-5      2023-11-28 [1] CRAN (R 4.3.2)
##    utf8           1.2.4      2023-10-22 [1] CRAN (R 4.3.2)
##    vctrs          0.6.5      2023-12-01 [1] CRAN (R 4.3.2)
##    withr          3.0.0      2024-01-16 [1] CRAN (R 4.3.2)
##    xfun           0.41       2023-11-01 [1] CRAN (R 4.3.2)
##    xtable         1.8-4      2019-04-21 [1] CRAN (R 4.3.2)
##    yaml           2.3.8      2023-12-11 [1] CRAN (R 4.3.2)
## 
##  [1] C:/Users/Paolo/AppData/Local/R/win-library/4.3
##  [2] C:/Program Files/R/R-4.3.2/library
## 
##  D ── DLL MD5 mismatch, broken installation.
## 
## ──────────────────────────────────────────────────────────────────────────────
```
